# A dual functional Ti-Ga alloy: inhibiting biofilm formation and osteoclastogenesis differentiation via disturbing iron metabolism

**DOI:** 10.1186/s40824-023-00362-1

**Published:** 2023-03-29

**Authors:** Fupeng Li, Kai Huang, Jinbing Wang, Kai Yuan, Yiqi Yang, Yihao Liu, Xianhao Zhou, Keyu Kong, Tao Yang, Jian He, Chunjie Liu, Haiyong Ao, Fengxiang Liu, Qian Liu, Tingting Tang, Shengbing Yang

**Affiliations:** 1 Department of Orthopaedic Surgery, Shanghai Ninth People’s Hospital, Shanghai Key Laboratory of Orthopaedic Implants, Shanghai Jiao Tong University School of Medicine, Shanghai, 200011 China; 2 Department of Oral and Maxillofacial-Head and Neck Oncology, Shanghai Ninth People’s Hospital, College of Stomatology, National Center for Stomatology, National Clinical Research Center for Oral Diseases, Shanghai Key Laboratory of Stomatology, Shanghai Jiao Tong University School of Medicine, Shanghai Jiao Tong University, Shanghai Research Institute of Stomatology, Shanghai, 200011 China; 3 Department of Materials Science and Engineering, Hong Kong Institute for Advanced Study, College of Science and Engineering, City University of Hong Kong, Hong Kong, China; 4 M-Duke Medical Technology (Shanghai) Co., Ltd, Shanghai, Shanghai China; 5 Jiangxi Key Laboratory of Nanobiomaterials & School of Materials Science and Engineering, East China Jiaotong University, Nanchang, 330000 China; 6 Department of laboratory medicine, Ren Ji Hospital, Shanghai Jiao tong university school of medicine, Shanghai, 200127 China

**Keywords:** Ti-Ga alloy, Anti-biofilm, Implant-associated infections, Bacterial adhesion

## Abstract

**Background:**

Although biomedical implants have been widely used in orthopedic treatments, two major clinical challenges remain to be solved, one is the bacterial infection resulting in biofilm formation, and the other is aseptic loosening during implantation due to over-activated osteoclastogenesis. These factors can cause many clinical issues and even lead to implant failure. Thus, it is necessary to endow implants with antibiofilm and aseptic loosening-prevention properties, to facilitate the integration between implants and bone tissues for successful implantation. To achieve this goal, this study aimed to develop a biocompatible titanium alloy with antibiofilm and anti-aseptic loosening dual function by utilizing gallium (Ga) as a component.

**Methods:**

A series of Ti-Ga alloys were prepared. We examined the Ga content, Ga distribution, hardness, tensile strength, biocompatibility, and anti-biofilm performance in vitro and in vivo. We also explored how Ga^3+^ ions inhibited the biofilm formation of *Staphylococcus aureus* (*S. aureus*) and *Escherichia coli* (*E. coli*) and osteoclast differentiation.

**Results:**

The alloy exhibited outstanding antibiofilm properties against both *S. aureus* and *E. coli *in vitro and decent antibiofilm performance against *S. aureus *in vivo. The proteomics results demonstrated that Ga^3+^ ions could disturb the bacterial Fe metabolism of both *S. aureus* and *E. coli*, inhibiting bacterial biofilm formation. In addition, Ti-Ga alloys could inhibit receptor activator of nuclear factor-κB ligand (RANKL)-dependent osteoclast differentiation and function by targeting iron metabolism, then suppressing the activation of the NF-κB signaling pathway, thus, showing their potential to prevent aseptic loosening.

**Conclusion:**

This study provides an advanced Ti-Ga alloy that can be used as a promising orthopedic implant raw material for various clinical scenarios. This work also revealed that iron metabolism is the common target of Ga^3+^ ions to inhibit biofilm formation and osteoclast differentiation.

**Supplementary Information:**

The online version contains supplementary material available at 10.1186/s40824-023-00362-1.

## Background

Medically implanted biomaterials play a vital role in the success of orthopedic procedures. Currently, pure titanium and titanium alloys are the commonly used materials for permanent implants in contact with bone due to their excellent mechanical properties, biological inertia, and corrosion resistance [1, 2]. Such implants have helped thousands of patients and improved their quality of life. However, due to the increased osteoclast activity, pure titanium, and its alloys face two intractable problems: biofilm infections and bone resorption around the implant [3]. Bacteria easily form biofilms on implants because local tissue reactions could be induced by foreign biomaterials, which contribute to the formation of a local immunosuppressive region, predisposing for bacterial proliferation, while biomaterials themselves provide substrates for bacterial attachment [4]. Once bacteria adhere to the material surface, biofilm formation is formally initiated through proliferation, maturation, and dissemination. Biofilm formation has become one of the crucial reasons for the problematic treatment of chronic and recrudescent orthopedic plant infections. It results in disability, reduces the quality of life, and brings an economic burden [5]. Current clinical therapies for biofilm infections include enhanced antibiotic use and implant replacement [6]. However, high antibiotics are likely to induce systemic side effects, and a second revision is undoubtedly a physical and mental disaster for patients. Other possible strategies include synergistic therapy with antimicrobial peptides and classical antibiotics, biofilm inhibitors, bacteriophages, and nanomaterials [7–9]. Nevertheless, their treatment efficacy in vivo remains to be assessed.

The loosening of implants caused by osteolysis is another factor limiting the therapeutic effects of titanium alloy implants [10]. The local inflammatory environment induced by titanium particles and the biofilm infection promotes the expression of macrophage colony-stimulating factor (M-CSF) and RANKL, leading to osteoclast activation and subsequent osteolysis [11]. Notably, wear particles can aggravate biofilm infection and enhance inflammation by inhibiting immune responses [12]. Targeting the overactivation of osteoclasts to ameliorate wear debris-induced osteolysis around prostheses is of great value to the clinic. The recent development of novel materials provides opportunities to address this issue. Sun successfully reduced ultra-high molecular weight polyethylene particle-induced osteolysis using niobium carbide [13]. Chitosan-derived nitrogen-doped carbon dots and Zn–Ag alloys also achieved satisfactory effects [14, 15]. However, in vivo biocompatibility and mechanical properties of the materials require further improvement. Thus, there is an urgent need to develop an implant material that can inhibit biofilm formation and block osteoclast activation to solve the two critical transplant-related issues.

Ga^3+^ ions have recently been shown to inhibit the activity of corresponding biofilm formation and block osteoclast activation [16, 17]. Ga^3+^ ions are believed to exert antimicrobial effects by competitively replacing iron with highly similar chemical structures. The iron-dependent bacterial enzymes become dysfunctional as Ga^3+^ ions uptake fails to be reduced to bivalent iron, which induces bacterial death [18, 19]. Our previous work also demonstrated that Ga^3+^ ions have excellent scavenging effects on MRSA and *E. coli* [20]. However, the mechanism by which Ga^3+^ ions inhibit bacterial biofilm formation remains unclear, limiting their further biomedical applications. Ga^3+^ ions have been widely reported to inhibit osteoclast activation, and Ga-based agents have been used to treat hypercalcemia in clinical settings [21, 22]. As there are few studies on the specific mechanism underlying the effect of Ga^3+^ ions on osteoclast inhibition, this needs to be further explored.

**Scheme 1:**
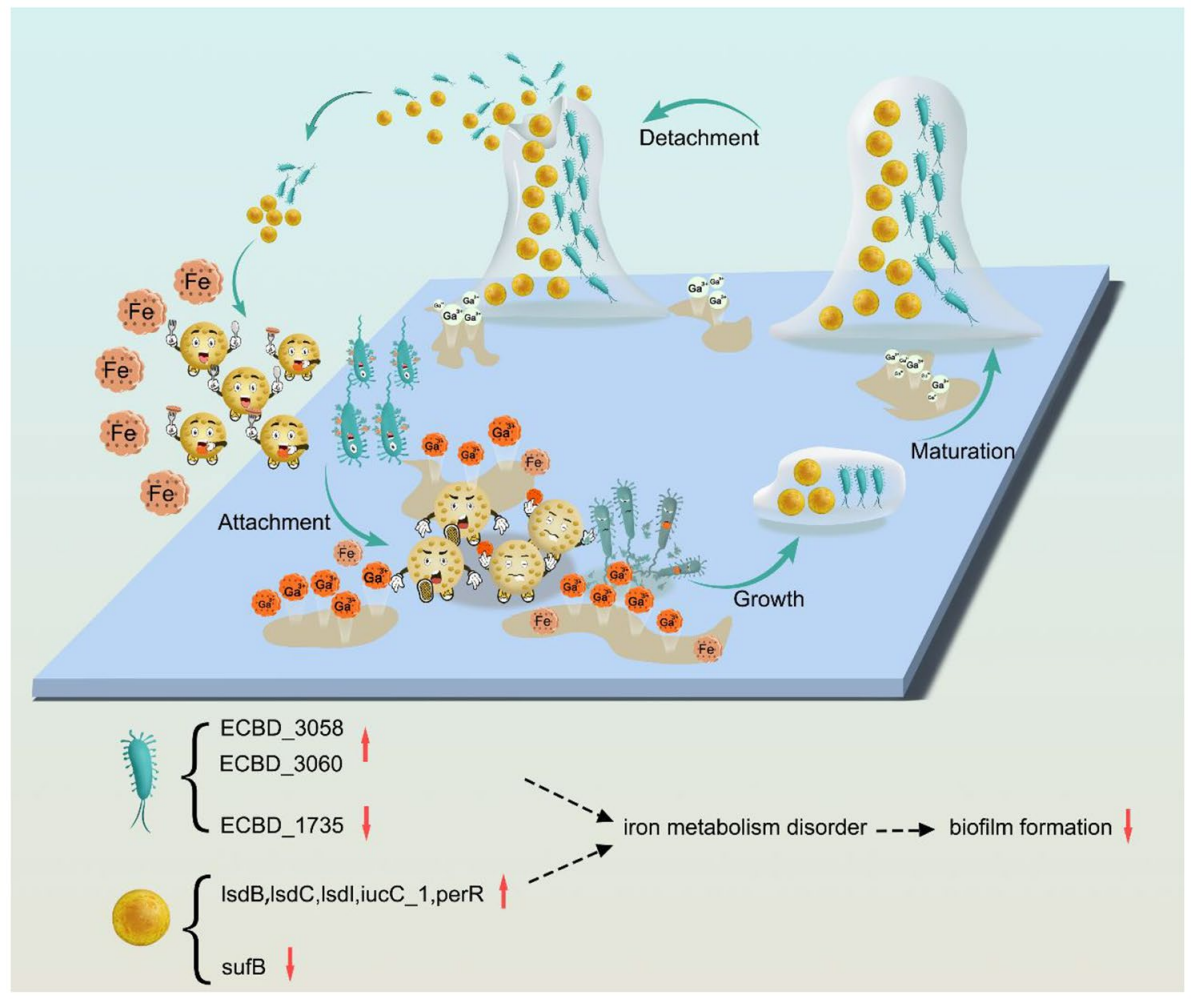
Ti-Ga alloy against *S. aureus* and *E. coli* biofilm formation by disturbing iron metabolism

In this study, we utilized Ga to improve the traditional Titanium alloy property of inhibiting biofilm and osteoclast activation, identified that Ga^3+^ ions significantly disturbed *S. aureus* and *E. coli* Fe metabolism based on proteomics analysis, and discovered that Ga^3+^ ions could significantly inhibit RANKL-dependent osteoclast differentiation by inhibiting iron uptake and the NF-κB signaling pathway (Scheme 1). Biofilm infection models in vivo and in vitro were taken to explore the bacterial burden associated with the Ti-Ga alloy group compared with that associated with the pure Ti group, using the plate counting method, scanning electron microscopy, confocal microscopy, crystal violet staining, and an *S. aureus* Xen 29 infection mice model. The obtained results confirmed that Ga^3+^ ions exerted outstanding anti-biofilm activity by intervening in bacterial iron metabolism. Simultaneously, the alloy showed excellent inhibiting osteoclast activation capability. In summary, we identified iron metabolism could be a potential target for inhibiting biofilm formation and osteoclastogenesis differentiation, and designed a titanium-Ga-based biomedical material that could not only impede biofilm formation but also block osteoclast activation, shedding new light on solving the clinical issues associated with implant allocation.

## Materials and methods

### Preparation of Ga-based alloys

The vacuum electric arc furnace was used for alloy casting. Different amounts of Ga were initially added, 0, 2.5, 5, 10, and 20%, respectively, and were denoted as 0, 2.5, 5, 10, 20 Ga group. The raw metals were heated to a melting state and kept melting for 5 min with electromagnetic stirring. Then, the samples were cooled to room temperature, and obtained the alloy ingot. Finally, the alloy ingot was shaped into a specific disc for further use. Analysis of X-rays diffraction (XRD), microhardness, atom probe tomography (APT), tensile test, scanning electron microscope (SEM), and inductively coupled plasma optical emission spectroscopy (ICP) were performed to characterize the synthesized Ti-Ga alloy.

### Cell culture and viability assays

Primary bone mesenchymal stem cells (BMSC) were isolated from the femur and tibia of 4-week-old male SD rats and cultured in α-MEM medium (HyClone, USA) containing 10% fetal bovine serum (Gibco, USA) and 1% penicillin-streptomycin solution (HyClone). Primary bone marrow-derived macrophages (BMMs) were isolated from the femur and tibia of 4-week-old male C57BL/6J mice and cultured in α-MEM containing 10% fetal bovine serum (FBS) and M-CSF (30 ng mL^− 1^). Cells were maintained at 37 ℃ in a humidified atmosphere containing 5% CO_2_. The cytotoxicity of the Ti-Ga alloy in BMSC was determined using the CCK-8 assay (Beyotime, China). Cells were seeded into 96-well plates at 8000 cells per well. After adhesion for 12 h, cells were treated with alloy extracts for 1, 3, 5, and 7 days. The optical density (OD) at 450 nm was measured using a multifunctional microplate reader (Tecan, M200pro, Switzerland). To further observe the effect of the Ti-Ga alloy on BMSC activity, cytoskeletal staining (phalloidin for cytoskeletal labeling, DAPI for nuclear counterstaining) was performed. Cell survival and spreading were observed using a laser confocal microscope (CLCSM, Leica, Germany).

### Global protein expression profiles of ***S. aureus*** (ATCC 25,923) and ***E. coli*** (ATCC 25,922) treated by Ga^3+^ ions

TMT-labelled quantitative proteomics analysis was based on data-dependent acquisition-based mass spectrometry (MS). 1 mL *S. aureus* or *E. coli* suspensions containing 10^7^ cells were added to the centrifuge tube. Then Ga(NO_3_)_3_ was introduced into the suspension. Then, proteins were digested with SDT buffer (4% SDS, 100 mM Tris-HCl, pH 7.6). The peptide mixture of each sample was labelled with a TMT reagent (Thermo Scientific, Massachusetts, USA) and fractionated with a Peptide Fractionation Kit (Thermo Scientific, Massachusetts, USA). Then, an Easy nLC-coupled Q Exactive mass spectrometer (Thermo Scientific, Massachusetts, USA) was operated in positive ion mode. MS data were acquired via a data-dependent top 10 method. The MS raw data were processed by the MASCOT engine (Matrix Science, London, UK; version 2.2), and the identification of proteins was performed by matching the data with the reference public database (*S. aureus* 133,769, UniProt; *E. coli* 4172, Uniprot). A fold change of protein (FC) ≥ 1.2-fold or ≤ 0.83 and *P* value < 0.05 were considered differentially expressed proteins.

### ***In vitro*** anti-adherence assays

*E. coli* (ATCC 25,922) was selected for adhesion to the alloy surfaces. A volume of 2 mL of the bacterial suspensions at a concentration of 1 × 10^8^ CFU (colony forming unit) mL^− 1^ in TSB medium was added to wells containing Ti and Ti-Ga alloys and incubated at 37 °C for 2 h. Next, the alloys were gently washed with sterile phosphate-buffered saline (PBS) three times to remove loosely adherent bacteria. The adherent bacteria on the alloys were fixed using 5% paraformaldehyde for 15 min. LIVE/DEAD staining (Sigma, USA) was used to visualize alloy surface bacterial adherence. Ti and Ti-Ga alloys were placed in sterile tubes containing TSB culture solution (1 mL). An ultrasonic water washer (B3500S-MT, Branson Ultrasonics Co., Shanghai, China) was used to separate the bacteria adhering to the surface of the alloys at 50 Hz for 30 min, diluted with an ultrasonically treated bacterial suspension in a gradient. Next, 100 µL of the suspension was aspirated and spread uniformly on TSA culture plates. After incubation at 37 °C in a constant-temperature incubator for 24 h, the number of colonies on TSA plates was counted to evaluate the anti-adherence performance of the alloy surface.

### Evaluation of antimicrobial properties of material surfaces (spread plate method)

After sterilization with 75% ethyl alcohol for 24 h, the metal disks (Ti–Ga alloys or pure Ti) were placed into a 24-well plate, and the surface anti-biofilm properties of the alloys were evaluated using the spread plate method. One milliliter of a bacterial suspension at a concentration of 1 × 10^6^ CFU mL^− 1^ was added to a 24-well plate, and the bacterial suspension was co-cultured with the alloys for 24 h. Samples were collected and gently rinsed thrice with PBS. Subsequently, alloys were placed in sterile tubes containing TSB culture solution (1 mL), and an ultrasonic water washer (B3500S-MT, Branson Ultrasonics Co.) was used to separate the bacteria adhering to the surface of the alloys at 50 Hz for 30 min, diluted with an ultrasonically treated bacterial suspension in a gradient. Next, 100 µL of the suspension was aspirated and spread uniformly on TSA culture plates. After incubation at 37 °C in a constant-temperature incubator for 24 h, the number of colonies on TSA plates was counted to evaluate the antibacterial performance of the alloy surface.

### Live-dead biofilm fluorescent imaging

For CLSM, the metal discs were gently rinsed with PBS three times, and the biofilms on Ti and Ti-Ga discs were stained using the LIVE/DEAD kit (Sigma) in the dark for 30 min at room temperature. Finally, cells were imaged using confocal laser scanning microscopy to observe biofilm formation (CLCSM; Leica TCS SP8).

### SEM assay

For SEM, the Ti and Ti-Ga discs were gently washed with sterile PBS three times and fixed with 4% paraformaldehyde for 20 min at room temperature. Subsequently, gradient dehydration was performed. Finally, samples were observed using SEM.

### Crystal violet staining

For crystal violet staining, Ti and Ti-Ga alloys were collected and gently washed with PBS three times. Next, the biofilms were stained with 1% crystal violet for 15 min, and the Ti discs were rinsed twice with DI water to remove excess staining and solubilized with 96% ethanol. The OD at 595 nm was determined to measure biofilm mass using a microtiter plate reader (Tecan, M200pro, Switzerland).

### TRAP staining to assess mature osteoclasts

After cell counting, BMMs were seeded into 96-well plates at 8000 cells/well density and incubated overnight. The following day, the upper layer of the medium was aspirated and discarded. The α-MEM cell medium containing pure Ti or Ti–Ga alloy extract (containing 50 ng mL^− 1^ RANKL and 30 ng mL^− 1^ M-CSF) was added for induction, and the medium was changed every other day. On day 6, cells were fixed with 4% paraformaldehyde and stained using the TRAP staining kit (Sigma) before further observation under a light microscope (Olympus, IX71, Japan).

### Actin ring formation assay

BMMs were seeded into 96-well culture plates (8000 per/well), cultured in complete α-MEM, and treated with Ti and Ti-Ga alloy extracts in the presence of M-CSF and RANKL for 6 days. After 4% paraformaldehyde fixation, cells were stained with rhodamine-conjugated phalloidin for 1 h (Beyotime Biotechnology, China), 4ʹ,6-diamidino-2-phenylindole for 15 min (DAPI; Beyotime Biotechnology) and washed with PBS three times. Finally, actin rings were observed and imaged using a fluorescence microscope (Olympus, IX71, Japan).

### Osteoclast bone resorption study

Bovine bone slices were sterilized with 75% ethyl alcohol for 24 h and then soaked in PBS in a 96-well plate for another 24 h to remove residual ethylene oxide. BMMs (8000 cells/well) were seeded on bovine bone slices and incubated overnight. Cells were then cultured with Ti and Ti-Ga alloy extract in the presence of M-CSF (30 ng mL^− 1^) and RANKL (50 ng mL^− 1^) for 2 weeks. The culture medium was replaced every other day. After 2 weeks, the cells on the slices were removed using 0.25% EDTA-trypsin. The bone slices were dehydrated by gradient ethyl alcohol and then coated with gold for observation under a scanning electron microscope (SEM; HITACHI, S4800, Japan).

### Osteoclast-related gene expression PCR assay

BMMs were cultured in 6-well plates (2 × 10^5^ cells/well) overnight for adherence. Cells were then stimulated with the Ti-Ga alloy extract containing M-CSF (30 ng mL^− 1^) and RANKL (50 ng mL^− 1^) for 5 days. Total RNA from BMMs was isolated using a TRIzol reagent (TIANGEN, China), and 1 µg of total RNA template was transcribed into cDNA using the cDNA Synthesis SuperMix (Yeasen Biotechnology, Shanghai, China). Real-time PCR was performed on a real-time PCR platform (Applied Biosystems, ABI 7500, USA) using SYBR Green qPCR Master Mix (Bimake, USA). Measured genes were normalized to β-actin and calculated using the 2^−ΔΔCt^ method. The primers used are listed in Table 1.

**Table 1:** Primer Sequences for qRT-PCR

Gene	Forward Primer Sequence (5ʹ-3ʹ)	Reverse Primer Sequence (5ʹ-3ʹ)
Ctsk	GGACCCATCTCTGTGTCCAT	CCGAGCCAAGAGAGCATATC
Acp5	CACTCCCACCCTGAGATTTGT	CATCGTCTGCACGGTTCTG
C-Fos	CCAGTCAAGAGCATCAGCAA	AAGTAGTGCAGCCCGGAGTA
Nfatc1	TGCTCCTCCTCCTGCTGCTC	GCAGAAGGTGGAGGTGCAGC
CTR	CGGACTTTGACACAGCAGAA	AGCAGCAATCGACAAGGAGT

**Abbreviations**: Ctsk, cathepsin K; Acp5, acid phosphatase 5; C-Fos, proto-oncogene C-Fos; Nfatc1, nuclear factor of activated T-cells c1; CTR, calcitonin receptor.

### Intracellular iron analysis

FerroOrange working solution at a concentration of 1 µM was added, and cells were incubated at 37 ℃ in a 5% CO_2_ incubator. Cells were observed under a confocal microscope immediately after ferroorange staining was added.

### Intracellular ROS analysis

Cells were incubated in the dark at 37 °C in a serum-free medium containing 10 µM DCFH-DA (Beyotime, China) for 30 min. The cells were washed three times in a serum-free medium to remove extracellular DCFH-DA. Cells were immediately photographed and observed using a confocal microscope.

### Western blotting

BMMs were cultured in 6-well plates (5 × 10^5^ cells/well) overnight. Cells were treated with extracts of Ti and Ti-Ga alloys and subsequently cultured in the presence or absence of RANKL for 20 min. Later, cells were washed once with PBS and harvested in RIPA lysis buffer containing a protease inhibitor cocktail and a phosphatase inhibitor. Total protein concentration was determined using a BCA protein assay kit (Beyotime Biotechnology). Proteins (20 µg) were separated using SDS sulfate-polyacrylamide gel electrophoresis (SDS-PAGE) and transferred onto a polyvinylidene fluoride membrane (Millipore, USA). The membrane was blocked in 5% non-fat milk for 1 h, incubated in primary antibody solution overnight at 4 ℃, incubated with secondary antibody for 1 h at room temperature, and detected using an infrared imaging system (Odyssey, USA). The primary antibodies used were as follows: β-actin (Affinity, AF7018, USA, 1:5000), P65 (Cell Signaling Technology, USA, 8242, 1:1000), p-P65 (Cell Signaling Technology, 3033, 1:1000), and IκBα (Cell Signaling Technology, 4814, 1:1000).

### Immunofluorescent analysis of NF-κB

BMMs were cultured in glass-bottom dishes and induced with RANKL and Ti-Ga alloy extracts for 30 min. After treatment, cells were fixed in 4% paraformaldehyde for 30 min, permeabilized with 1% Triton X-100 in PBS for 10 min, and blocked with 1% BSA for 1 h. For P65 staining, cells were incubated with the P65 antibody (Affinity, AF0874, 1:250) overnight and secondary antibody for 1 h. For the actin cytoskeleton staining, cells were stained with fluorescein isothiocyanate (FITC)-phalloidin for 30 min. The nuclei were counterstained with DAPI for 5 min. Fluorescence images were captured using a confocal laser scanning microscope (CLCSM, Leica TCS SP8).

### Subcutaneous implantation in mice

We established a dorsum subcutaneous implantation model to evaluate the anti-biofilm properties and biocompatibility of Ti-Ga alloys in vivo. All experimental procedures were approved and performed by the guidelines of the Animal Ethics Committee of Shanghai Ninth People’s Hospital. Briefly, specific-pathogen-free (SPF) 6-week-old female Kunming (KM) mice were randomly assigned to six groups: CTR (injected with bacteria without implants), Ti (injected with bacteria with Ti alloy), Ti-2.5Ga (injected with bacteria with Ti-2.5Ga alloy), Ti-5Ga (injected with bacteria with Ti-5Ga alloy), Ti-10Ga (injected with bacteria with Ti-10Ga alloy), and Ti-20Ga (injected with bacteria with Ti-20Ga alloy). The mice were initially anesthetized using an intraperitoneal injection of 1% pentobarbital sodium (100 mg kg^− 1^), and the dorsal side of the leg was shaved with 2% iodine spread before the operation. Next, a deep subcutaneous pocket within the muscle was made through an approximately 1 cm longitudinal skin incision, where the sterile alloy was placed. An inoculum of Xen 29 (1 × 10^8^ CFU in 100 µL of PBS) was percutaneously pipetted onto the alloy surface after the closure of the incision using a microsyringe.

### Real-time monitoring of bacterial growth using ***in vivo*** bioluminescence imaging

Bioluminescent *S. aureus* Xen 29 was selected as the bacterium for in vivo implant-related infection. The bacterial burden was monitored using fluorescence intensity. After anesthesia, bioluminescence was recorded using an IVIS Spectrum® imaging system (Caliper, PerkinElmer Company, Alameda, CA, USA) at the indicated time points.

### Microbiological evaluation

After the mice were sacrificed on days 7 and 14 post-surgery, the implanted alloys were collected and immediately placed in sterile centrifuge tubes containing 1 mL PBS. Bacteria adhering to the implant were collected using the ultrasonic water washer described above. The suspension was collected for a serial (10-fold) dilution. Antibacterial efficiency was analyzed using the spread plate method, as described above.

### Biofilm inhibition ***in vivo***

Animals were sacrificed on day 4 post-surgery, and the alloys were gently removed and rinsed with PBS twice. Alloys were then prepared for CLSM and SEM as described above.

### Histopathological evaluation

Briefly, the animals were sacrificed on days 7 and 14 post-surgery. The alloys were removed, and the surrounding muscles were carefully dissected from the femur. Excised soft tissue was immediately embedded into 4% neutral-buffered paraformaldehyde and subjected to histological analysis using H&E and Giemsa staining.

## Results

### Characterization of Ti-Ga alloy

**Fig. 1:**
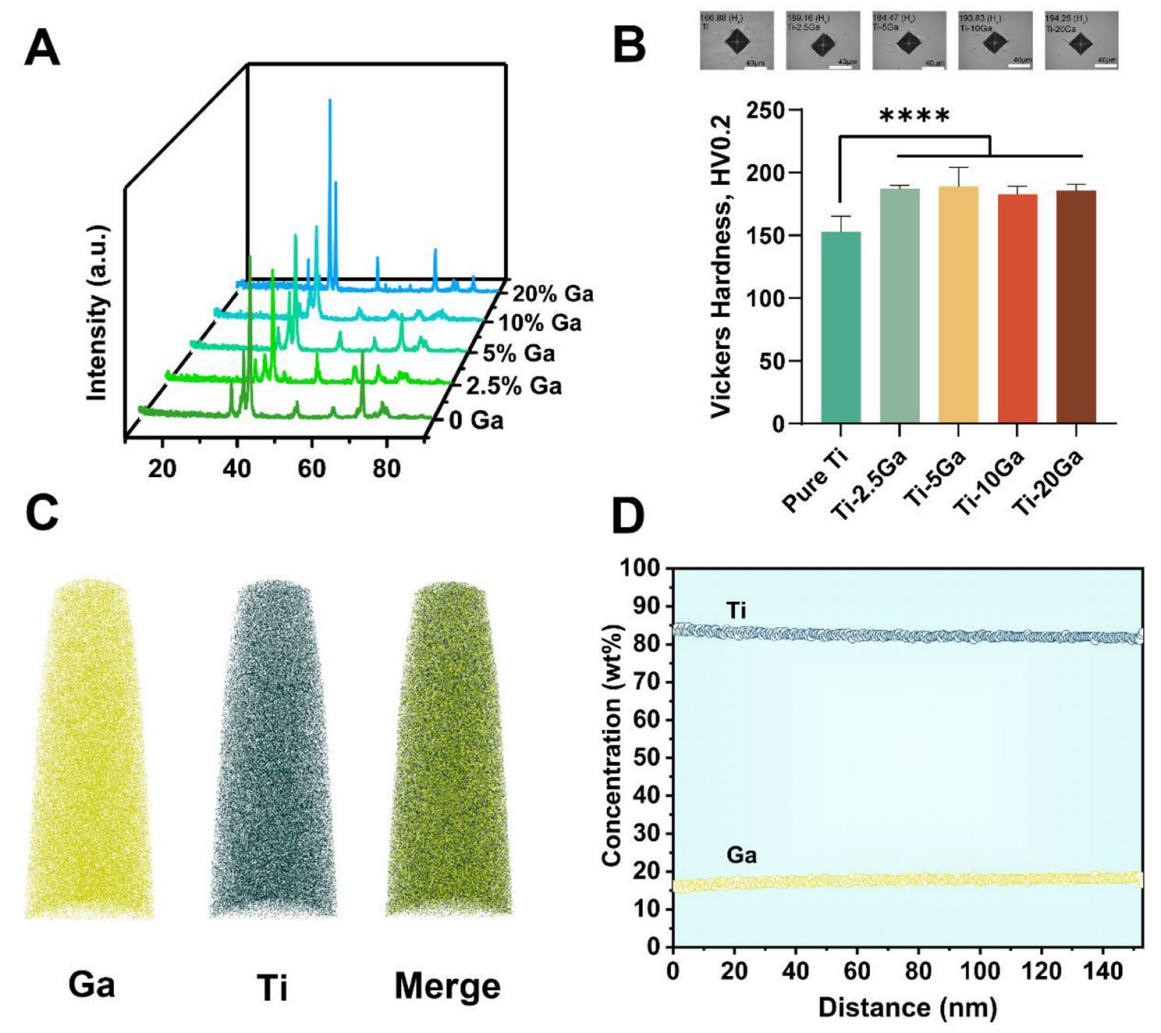
Characterization of synthesized Ti-Ga alloys. **(A)** Broaden XRD patterns of Ti-Ga alloys by adding 0, 2.5, 5, 10, and 20% Ga when initially casting. **(B)** Vickers hardness of pure Ti and Ti-Ga alloys. **(C)** APT characterization of the elemental partitioning of the denoted 20% Ga group. **(D)** Surface element distribution of the denoted 20% Ga group. (****indicates *p* < 0.0001 compared with the pure Ti group)

In this work, Ga was utilized to overcome the shortage of traditional titanium alloy. The primary objective was to make Ga evenly distributed in titanium alloy for further utilization. Firstly, XRD patterns of Ti-Ga alloys indicated that the synthesized samples (2.5%, 5%, 10% content groups) are single α phase and the sample of 20% content group is α + α2 phase (Fig. 1A) [23]. Then, we tested the microhardness of pure titanium and Ti-Ga alloy. The hardness of 2.5%, 5%, 10%, and 20% Ga containing Ti-alloy was slightly higher than pure titanium alloy (Fig. 1B), indicating improved mechanical properties of Ti-Ga alloy. Finally, we analyzed the denoted 20% content Ga sample’s gallium distribution using three-dimensional atom probe tomography (3D-APT), considering its highest Ga content. It was observed that Ga (yellow) and Ti (blackish green) were uniformly distributed in the alloy matrix (Fig. 1C). The concentration of surface Ga was relatively stable and maintained at about 17% percentage (Fig. 1D). From the observations above, we can see that Ga is homogeneously distributed in our developed Ti-Ga alloy, which is significant in exerting the anti-biofilm and inhibiting the osteoclastogenesis differentiation effect of Ga. Figures S1-S2 showed the yield strength (YS) of Ti-Ga alloys before and after immersion in simulated body fluid (SBF) for 8 weeks. With the elevation of gallium content, the YS of Ti-Ga alloy increases obviously, indicating that gallium improved the mechanical property of pure Ti. When the content of gallium reaches 20%, the YS of Ti-Ga alloy reaches 877.9 MPa, which is much higher than that of pure Ti (240–550 MPa) [24, 25]. In addition, after 8 weeks of immersion, the mechanical properties of Ti-Ga alloys have almost no change. The YS of Ti-20Ga only decreased from 877.9 MPa to 870.8 MPa. It’s of great significance for implants to be applied in vivo. We also explored the surface morphology of alloys after immersion. As was shown in Figure S3, no apparent changes were observed after immersion.

### Anti-biofilm activity of Ti-Ga alloy ***in vitro***

**Fig. 2:**
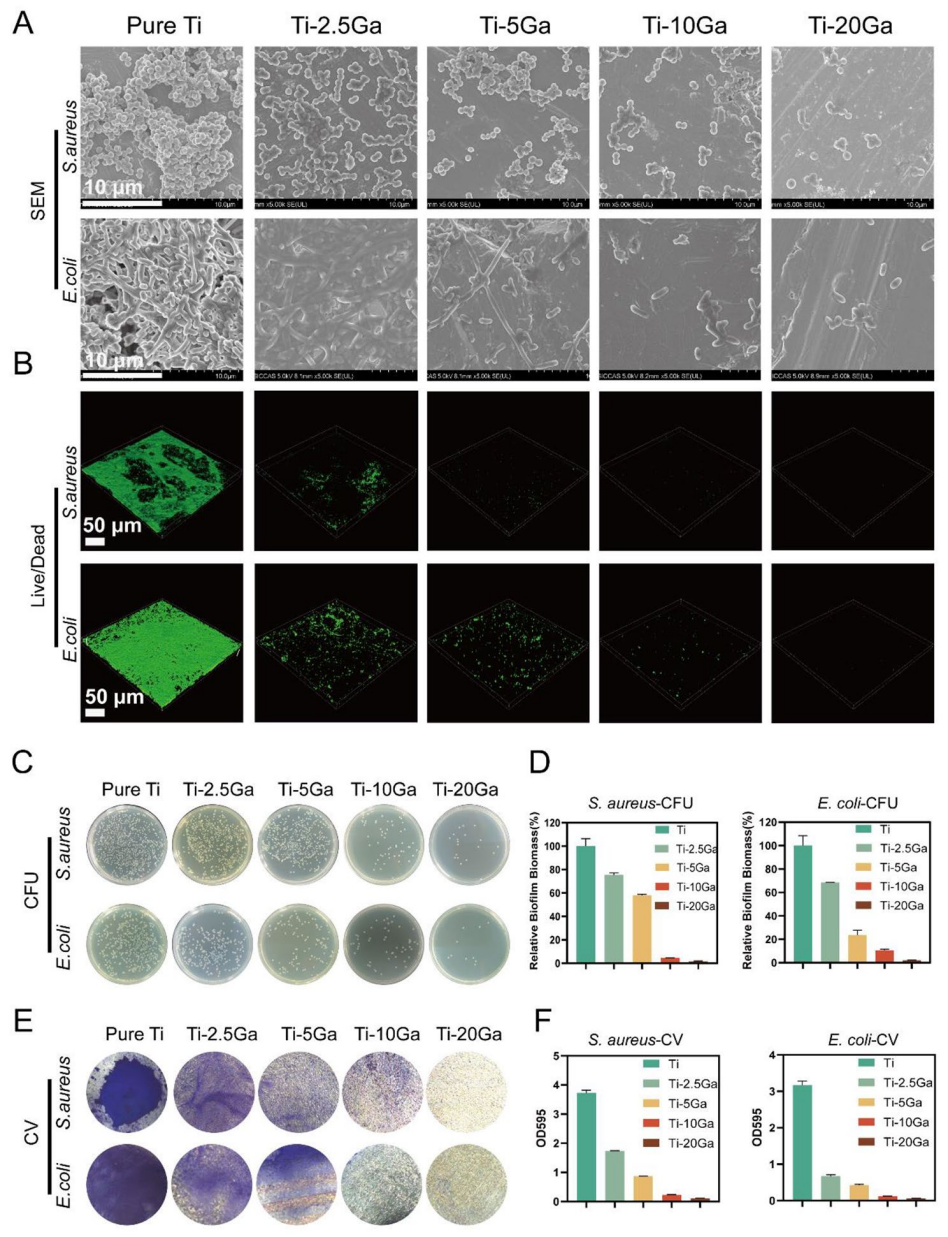
Biofilm-resistant performance of Ti-Ga alloy. **(A)** SEM images of biofilm formed by *S. aureus* and *E. coli* after different treatments. **(B)** Confocal 3D images of biofilm of *S. aureus* and *E. coli*. **(C)** Representative images of bacterial colonies from biofilms on the surfaces of alloys. **(D)** Relative amounts of *S. aureus* and *E. coli* biofilm biomass. **(E)** Biofilms treated with different groups of alloys for 24 h and stained with 1% crystal violet. **(F)** Quantification of biofilm formation of *S. aureus* and *E. coli* by using a microtiter plate reader

The Ga release profile of the Ti-Ga alloy was also determined in this study. A rapid release of Ga ions was detected within 15 days. After that, the release speed of Ga slowed down, and the accumulative release amount was 2.26 µM at the end time point of 60 days (**Figure S4**). Crystal violet staining was used to confirm the antibiofilm ability of Ga ions (**Figures S5 and S6**). Gallium ion at 1 µM could reduce *S.aureus* adhesion and prevent biofilm formation. At the same time, the MBIC of the Ga ion for *E. coli* was 0.8 µM. Based on the antibacterial performance of Ga^3+^ ions, the Ti-Ga alloy has an intervention potentiality on the biofilm formation of *S. aureus* and *E. coli* on biomaterial surfaces. SEM, confocal microscopy, plate counting, and crystal violet staining were performed to further evaluate the inhibitory effect of the Ti-Ga alloy on biofilm formation. SEM images indicated that bacteria interlinked closely and formed a dense and sophisticated structure, namely a biofilm, on the Ti surface. However, only sparse bacteria could be seen in the Ti-Ga group, suggesting that the Ti-Ga alloy disrupted biofilm formation (Fig. 2A). Three-dimensional confocal images further indicated that the pure Ti group was coated with large amounts of viable bacteria in a well-structured community. In contrast, almost no live bacteria existed on the surface of the Ti-20Ga alloy (Fig. 2B). The relative biofilm biomass was 100% ± 6.37% for *S. aureus* and 100% ± 8.4% for *E. coli* in the pure Ti group, which decreased with the increase in the Ga content of Ti-Ga alloy, leaving only 1.66% ± 0.29% and 2.03% ± 0.18% in the Ti-20Ga group for *S. aureus* and *E. coli*, respectively (Figs. 2C and D). Additionally, crystal violet staining and the corresponding OD values supported this finding in Figs. 2E F.

### ***S. aureus*** iron metabolism disorder following Ga treatment

**Fig. 3:**
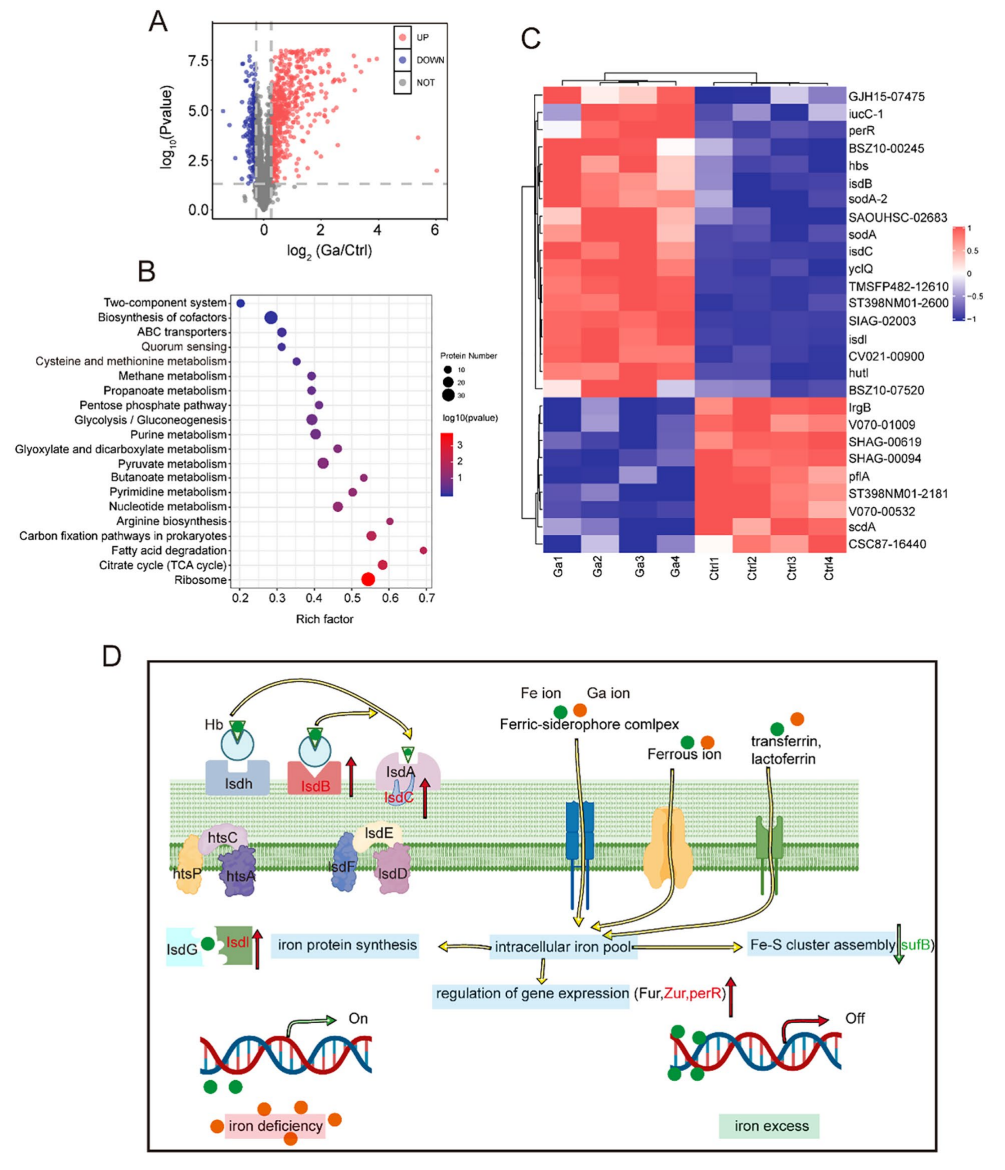
Proteomic revealed iron disorder of *S. aureus* after Ga(III) treatment. **(A)** Volcant plot. **(B)** Kegg enrichment. **(C)** Heat map of hierarchical clustering analysis of the iron metabolism related proteins after Ga treatment. **(D)** Possible mechanism of Ga disrupting *S. aureus* iron metabolism

Iron is required for bacterial survival, growth, proliferation, and pathogenesis, which participate in DNA synthesis and energy metabolism as a cofactor. According to our previous work, Ga-based compounds could remove *S. aureus* biofilm [26]. Therefore, we employed proteomics analysis to explore how Ga^3+^ ions affected iron metabolism. A total of 768 differentially expressed proteins (DEPs) of *S. aureus* were identified between the Ga^3+^ ions treatment group and the control (CTR) group, among which 227 were downregulated (fold change < 0.83, *p* < 0.05) and 541 were upregulated (fold change > 1.2, *p* < 0.05; Fig. 3A). Next, the functions of these DEPs were further summarized using a KEGG pathway analysis. As shown in Fig. 3B, the enrichment analysis indicated that cysteine and methionine metabolism, quorum sensing, and citrate cycle are mainly associated with the identified *S. aureus* proteins. Alterations in DEPs involved in iron metabolism were illustrated via a heat map (Fig. 3C). As was shown in **Figure S7**, proteins participating in iron uptake were significantly upregulated, especially siderophore synthase, responsible for siderophore synthesis. On the other hand, proteins involved in the assembly of the Fe-S cluster, which occurred in iron-replete conditions, were down-regulated. Hence, we summarized the potential mechanism of Ga^3+^ ions inhibiting biofilm formation: Ga^3+^ ions disturbed the metabolism of iron and caused an iron metabolic disorder state of *S. aureus*, then inhibited biofilm formation.

### ***E. coli*** iron metabolism disorder and biofilm formation-related proteins expression changes following Ga^3+^ ions treatment

**Fig. 4:**
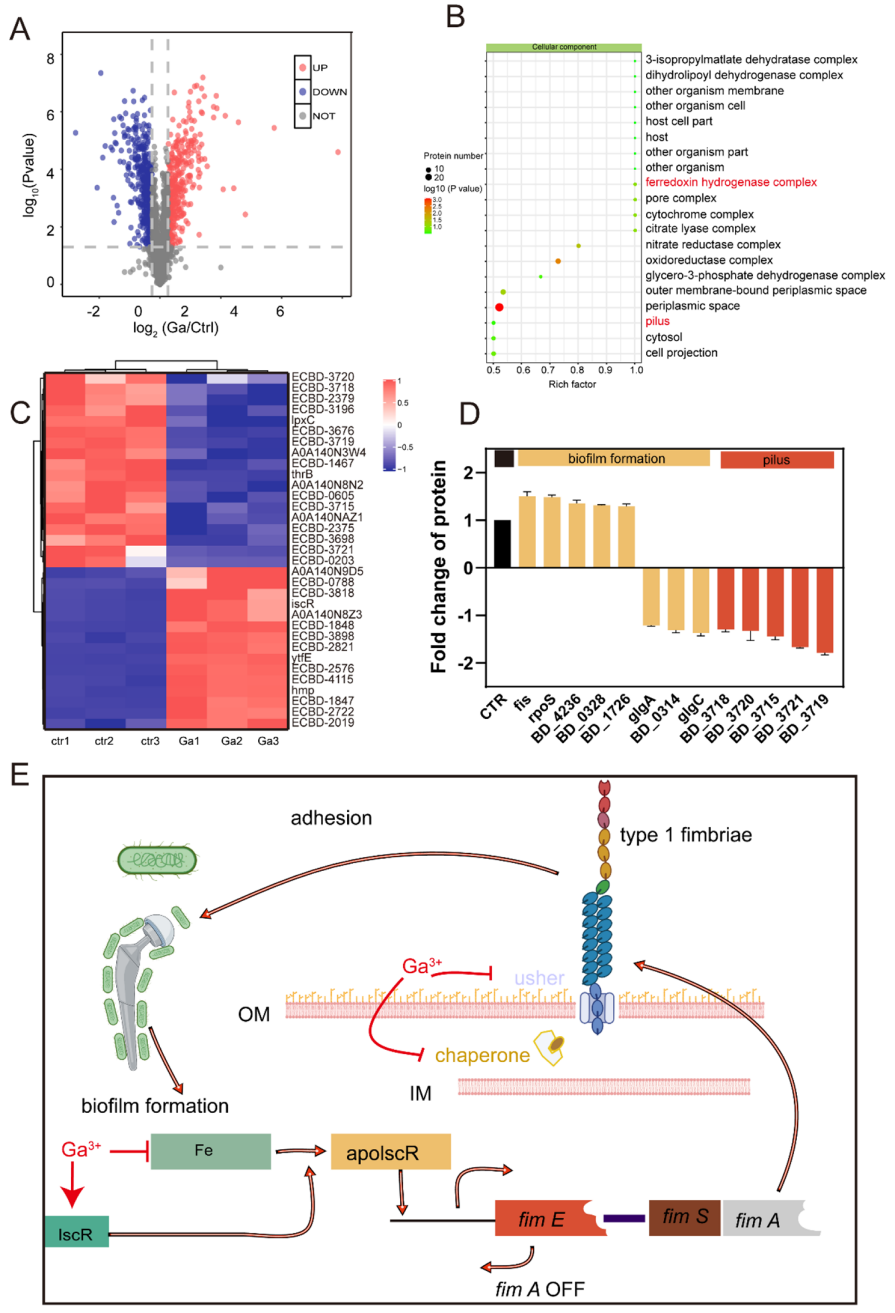
Decreased attachment capacity of *E. coli* meditated by iron deficiency after Ga treatment. **(A)** Volcant plot. **(B)** GO enrichment analysis (Cellular component) of differentially expressed proteins after Ga treatment. **(C)** Heat map of hierarchical clustering analysis of the differentially expressed proteins after Ga treatment. **(D)** Quantitative analysis of type 1 fimbriae assembly related proteins of *E. coli*. **(E)** The possible mechanism of Ga disrupting *E. coli* biofilm formation at the adhesion stage

Firstly, proteomics analysis was taken to explore how Ga affected iron metabolism. As for *E. coli*, 358 proteins were upregulated (fold change > 1.2, *p* < 0.05), and 404 proteins were significantly downregulated (fold change < 0.83, *p* < 0.05; Fig. 4A). The result of kegg enrichment reflected iron metabolism disorder as the pathway and biosynthesis of siderophore group nonribosomal peptides were enriched (Figure S8). A comparison of the main biological process and molecular functions of differentially expressed proteins is given in **Figures S9** and **S10**. Proteins participating in intracellular iron ion homeostasis and iron homeostasis biological process were significantly enriched. As for the molecular function part, proteins serving as Fe-S cluster assembly were enriched and marked in red. Finally, iron metabolism-related proteins in *E. coli* were quantitatively analyzed in Figure S11.

Biofilms are well-structured microbial communities composed of extracellular polymeric substances and embedded bacteria. The biological process of biofilm formation on the surface of biomaterials and tissues includes the initial adhesion stage, microcolony formation, biofilm maturation (bacteria proliferation and extracellular matrix secretion), and biofilm dispersion [27]. Attachment is the initial and critical step of biofilm formation [28]. Cell surface accessory structures, type 1 fimbriae (pilus), curli fimbriae, and cellulose play crucial roles in the attachment of *E. coli* to abiotic surfaces and host tissues [29]. Notably, the protein BcsE (ECBD-0203) responsible for cellulose production was downregulated by Ga^3+^ ions [30, 31]. While cellulose and curli negative regulator, Fis was upregulated (Fig. 4D). More importantly, we observed that proteins involved in biofilm formation at the adhesion stage, especially type 1 fimbriae assembled related proteins were significantly enriched (Fig. 4B). Type 1 fimbriae assembled through a chaperone-usher pathway. The mannose-binding domain protein (ECBD-3715), one component crucial for the adhesion of type 1 fimbriae, was significantly downregulated after Ga^3+^ ions treatment [32]. In addition, type 1 fimbriae negatively regulated proteins, and hemolysin expression modulating family proteins (ECBD-2019) was enhanced considerably by Ga^3+^ ions [33]. In contrast, proteins involved in the assembly of type 1 pilus, such as fimbrial protein (ECBD-3716), pili assembly chaperone (ECBD-3719), N-terminal, and fimbrial biogenesis outer membrane usher protein (ECBD-3718), were downregulated [34, 35]. The alterations of the above DEPs were visualized using a heat map (Fig. 4C), and the quantitative analysis of proteins for type 1 fimbriae assembly was presented in Fig. 4D. Based on our proteomic results, the Ga^3+^ ions treatment significantly disturbed the iron metabolism of *E. coli*. According to Wu, limited iron conditions can suppress type 1 fimbriae expression by activating the transcription of *fimE*, which downregulates the fim operon in *E. coli* [36]. Therefore, we preliminarily hypothesized that Ga could disrupt bacterial attachment by interfering with the corresponding functional proteins, and this action may be attributed to Fe limitations.

To further verify this hypothesis, confocal microscopy and CFU formation assays were used to visualize bacterial attachment. As shown in Figure S12, numerous bacteria adhered to the pure Ti alloy after 2 h, while fewer bacteria were observed on the surface of the Ti-20Ga alloy. The anti-adherence performance was consistent with the downregulation of biofilm formation proteins at the attachment stages. The Ga^3+^ ions treatment group demonstrated a similar bacterial attachment as observed in the Ti-20Ga group, thus, suggesting that the Ti-Ga alloy-induced bacterial adhesion inhibition resulted from the Ga^3+^ ions release from the alloy. Furthermore, the addition of Fe^3+^ reversed the bacterial burden induced by Ga^3+^ ions. Rescue experiments showed that the anti-adhesion effect of Ga^3+^ ions might be caused by iron deficiency. Finally, we illustrate our possible explanations for the anti-biofilm capacity of Ga^3+^ ions in Fig. 4E.

### Ti-Ga alloy can effectively inhibit RANKL-dependent osteoclast differentiation and bone resorption activity via disturbing iron metabolism

**Fig. 5:**
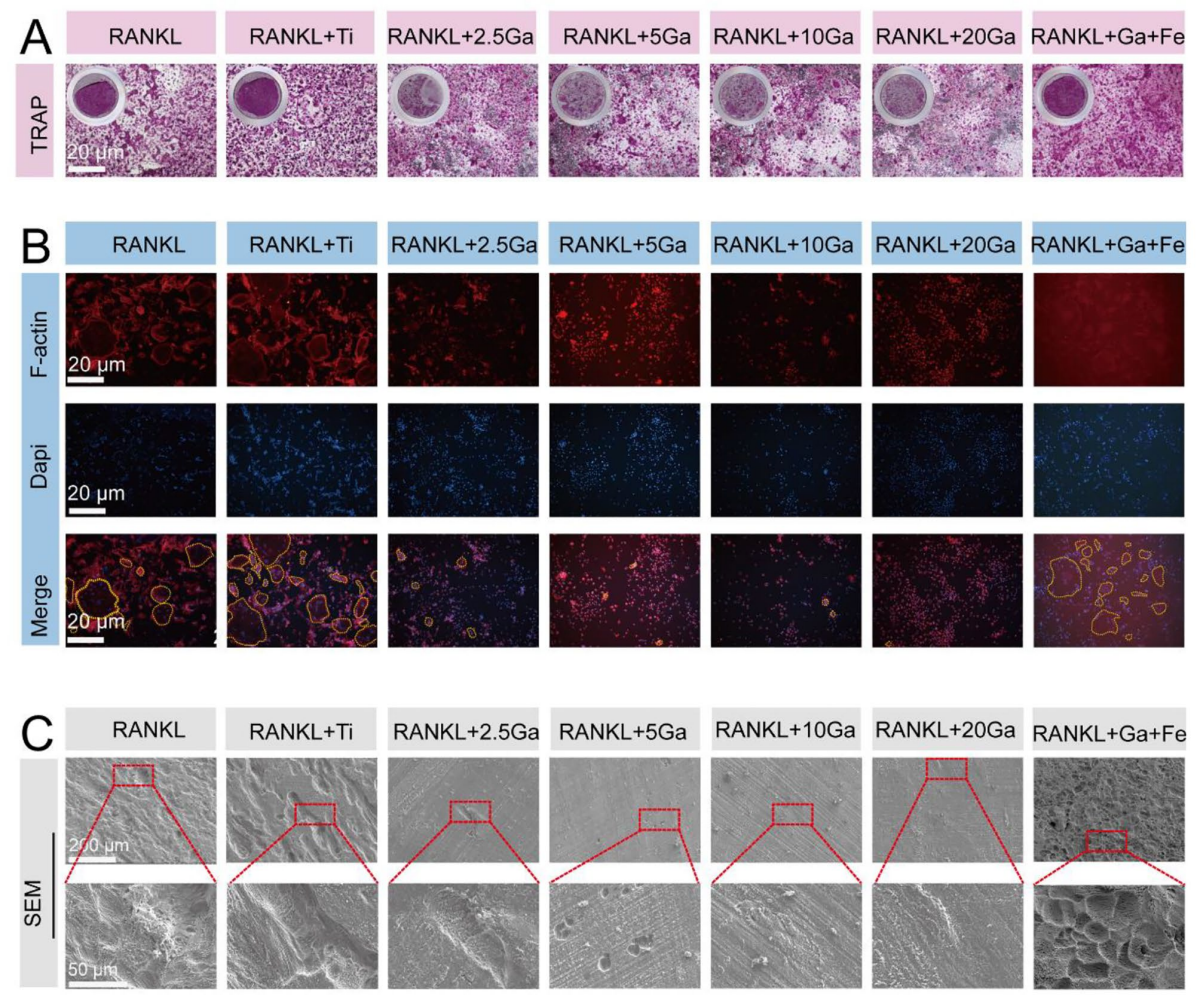
Ti-Ga alloys inhibited RANKL-dependent osteoclastogenesis in vitro targeting iron metabolism. **(A)** Representative images of TRAP staining. BMMs were stimulated with RANKL (50 ng mL^− 1^) in the absence or presence of extracts of Ti and Ti-Ga alloys, respectively. **(B)** Representative images of F-actin ring formation in osteoclasts treated with pure Ti and Ti-Ga alloy extracts, respectively. **(C)** Representative SEM images of bone resorption after treatment with extracts of pure Ti and Ti-Ga alloys respectively.

The effect of the Ti-Ga alloy on osteoclast formation was measured using Tartrate-Resistant Acid Phosphatase (TRAP) staining. Bone marrow-derived monocyte cells (BMMs) fuse to form mature multiple-nuclei osteoclasts. TRAP staining demonstrated that many positive multinucleated osteoclasts were formed following 5-day stimulation with RANKL. The addition of pure titanium extract exerted almost no effect on osteoclast formation. While the introduction of Ga^3+^ ions inhibited osteoclast formation, the TRAP-positive cell number and osteoclast area decreased in the low-content Ga alloy group. It is noteworthy that the inhibitory effect of Ga^3+^ ions on osteoclast differentiation and maturation can be rescued by iron, suggesting that Ga^3+^ ions may inhibit osteoclast differentiation and bone resorption through iron metabolism (Figs. 5A and S13). Intact F-actin rings are prerequisites for mature osteoclasts to resorb bone tissues [37]. Therefore, we examined the effect of a Ti-Ga alloy on the cytoskeleton of mature osteoclasts. As shown in Fig. 5B, the low content of Ti-Ga alloy inhibits the formation of the F-actin ring, whereas pure Ti alloy has no inhibitory effect on ring formation.

Next, we tested the effect of the Ti-Ga alloy on osteoclast bone resorption activity. After stimulation with RANKL, SEM showed obvious bone absorption cavities formed on the surface of fetal bovine bone slices in the CTR and pure Ti alloy groups. However, the Ti-Ga alloy inhibited the bone resorption function of osteoclasts. Figure 5 C showed that the Ti-Ga groups displayed fewer superficial bone resorption traps.

Quantitative real-time polymerase chain reaction (qRT-PCR) was further performed to evaluate the inhibitory effect of Ga^3+^ ions on osteoclast differentiation. As shown in Figure S14, the expression of osteoclast-specific genes, including *Acp5*, *Ctsk*, *C-Fos*, *CTR*, and *NFATC1* in BMMs was upregulated in the presence of RANKL, whereas these genes were downregulated by lower Ti-Ga alloy content [38]. To conclude, Ti-Ga alloy can effectively inhibit RANKL-dependent osteoclast differentiation and bone resorption activity via disturbing iron metabolism.

### Ti-Ga alloy disturbs iron metabolism and suppresses osteoclast differentiation via NF-κB signaling pathways meditated by ROS

**Fig. 6:**
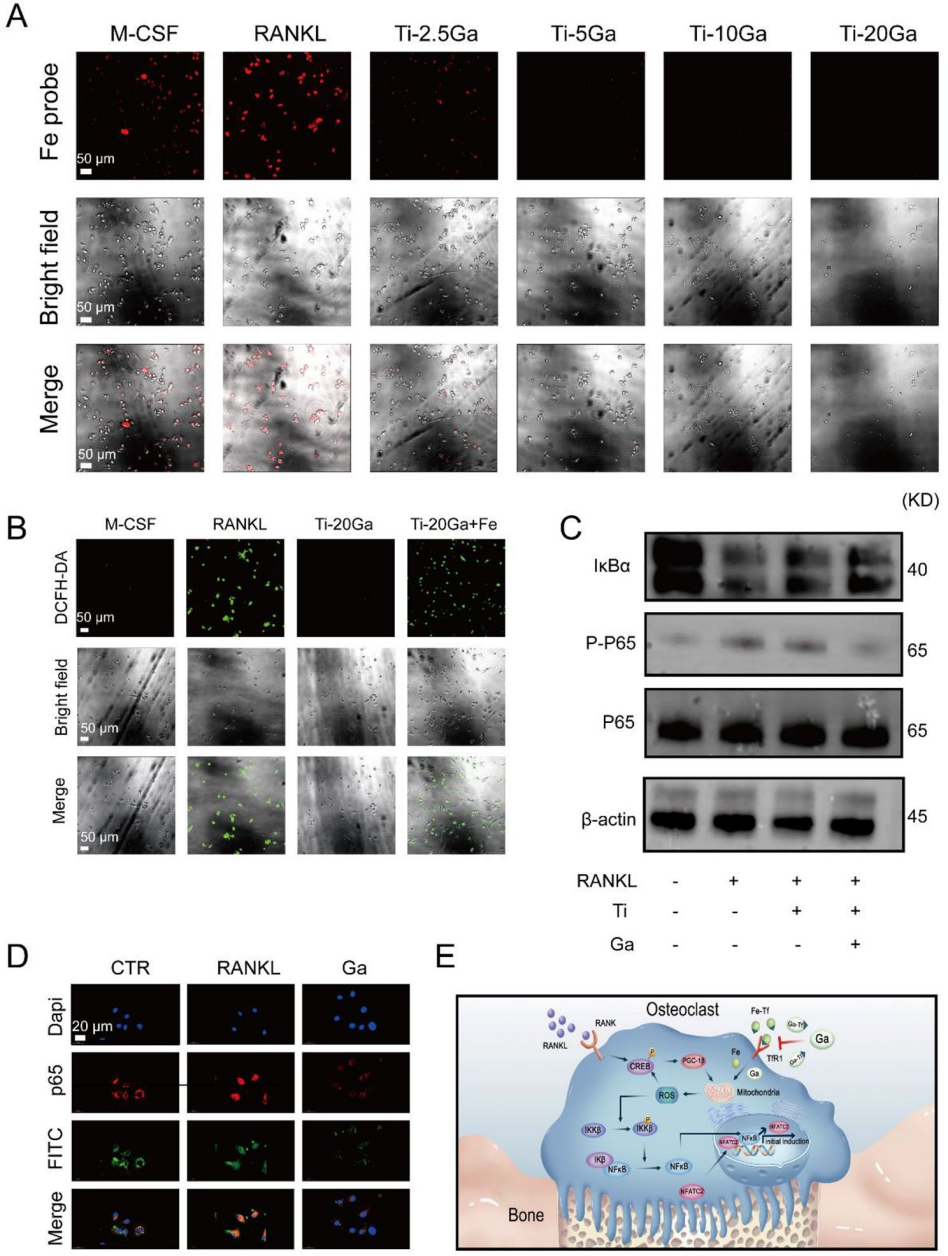
Ga^3+^ inhibited iron-induced osteoclastogenesis meditated by ROS via NF-κB signaling pathways. **(A)** Intracellular iron content after Ga^3+^ treatment. **(B)** Representative confocal images of intracellular ROS generation in BMMs. **(C)** BMMs cells were treated with Ti or Ti-Ga alloy extracts and RANKL (50 ng mL^− 1^). Cell lysates were subjected to Western blotting analysis for p-P65, P65 and IκB. **(D)** Immunofluorescence measured the translocation of P65 from the cytoplasm to the nucleus. **(E)** Molecular targets of Ga^3+^ action on osteoclast precursor cells differentiation

Although the inhibitory effect of Ga^3+^ ions on osteoclasts has been extensively studied, the mechanism by which Ga^3+^ ions inhibit osteoclast differentiation has rarely been reported. Iron metabolism is closely related to osteoclast differentiation: Patients with osteoporosis characterize by iron overload, and hepcidin can inhibit osteoclast differentiation by reducing intracellular iron content [39, 40]. Interestingly, Ga^3+^ ions exhibit an antibacterial role by interfering with bacterial iron metabolism. Whether Ga^3+^ ions inhibited osteoclastogenesis via iron and what targets downstream of iron attracted our attention. Our phenotypic experiments have demonstrated that iron could restore the inhibitory effect of Ga^3+^ ions on osteoclasts. Firstly, an iron probe staining experiment was adopted to observe the intracellular iron content of osteoclasts with Ga content varying after RANKL stimulation. The results indicated that more iron is required for osteoclast maturation and differentiation. After the introduction of Ga^3+^ ions, intracellular iron content decreased evidently in a dose-dependent manner, suggesting that Ga^3+^ ions interfered with iron metabolism in osteoclast precursor cells (Fig. 6A). Jia’s study showed that iron promotes osteoclast differentiation and bone resorption by producing ROS [41]. Therefore, we used a 2′,7′-dichlorofluorescein diacetate (DCFH-DA) probe to analyze the effect of Ga^3+^ ions on the expression of ROS, a downstream product of iron. As shown in Fig. 6B, intracellular ROS increased after RANKL activation. At the same time, ROS levels were down-regulated in the Ga^3+^ ions treatment group, and the re-introduction of iron restored intracellular ROS levels. This is consistent with the change in intracellular iron content, indicating that Ga^3+^ ions inhibit the expression of ROS signaling molecules by inhibiting iron. According to Ishii et al., iron uptake is required for efficient osteoclast maturation signaling via MAP kinase and IKK cascades downstream of the RANK [42]. Similarly, Wang et al. demonstrated that iron promotes osteoclast differentiation by producing ROS via the NF-κB signaling pathway [43]. To this end, we used immunofluorescence staining and Western blot to verify whether Ga^3+^ ions inhibited the activation of the NF-κB signaling pathway by disturbing iron metabolism. Western Blot results demonstrated that Ga^3+^ ions inhibited activation of the NF-κB signaling pathway (Fig. 6C). Immunofluorescence staining is presented in Fig. 6D. The translocation of P65 into the nucleus increased after adding RANKL to the iron-containing medium, while the introduction of Ga^3+^ ions successfully blocked it. The introduction of Ga^3+^ ions inhibited the RANKL-induced NF-κB signaling pathway activation in the iron-containing medium. These results suggested that Ga^3+^ ions inhibited osteoclast differentiation by interfering with osteoclast precursor cells. Dysregulation of iron metabolism further leads to the downregulation of ROS production and subsequent inhibition of NF-κB signaling activation. The possible molecular mechanism was presented in Fig. 6E.

### ***In vivo*** anti-biofilm activity

**Fig. 7:**
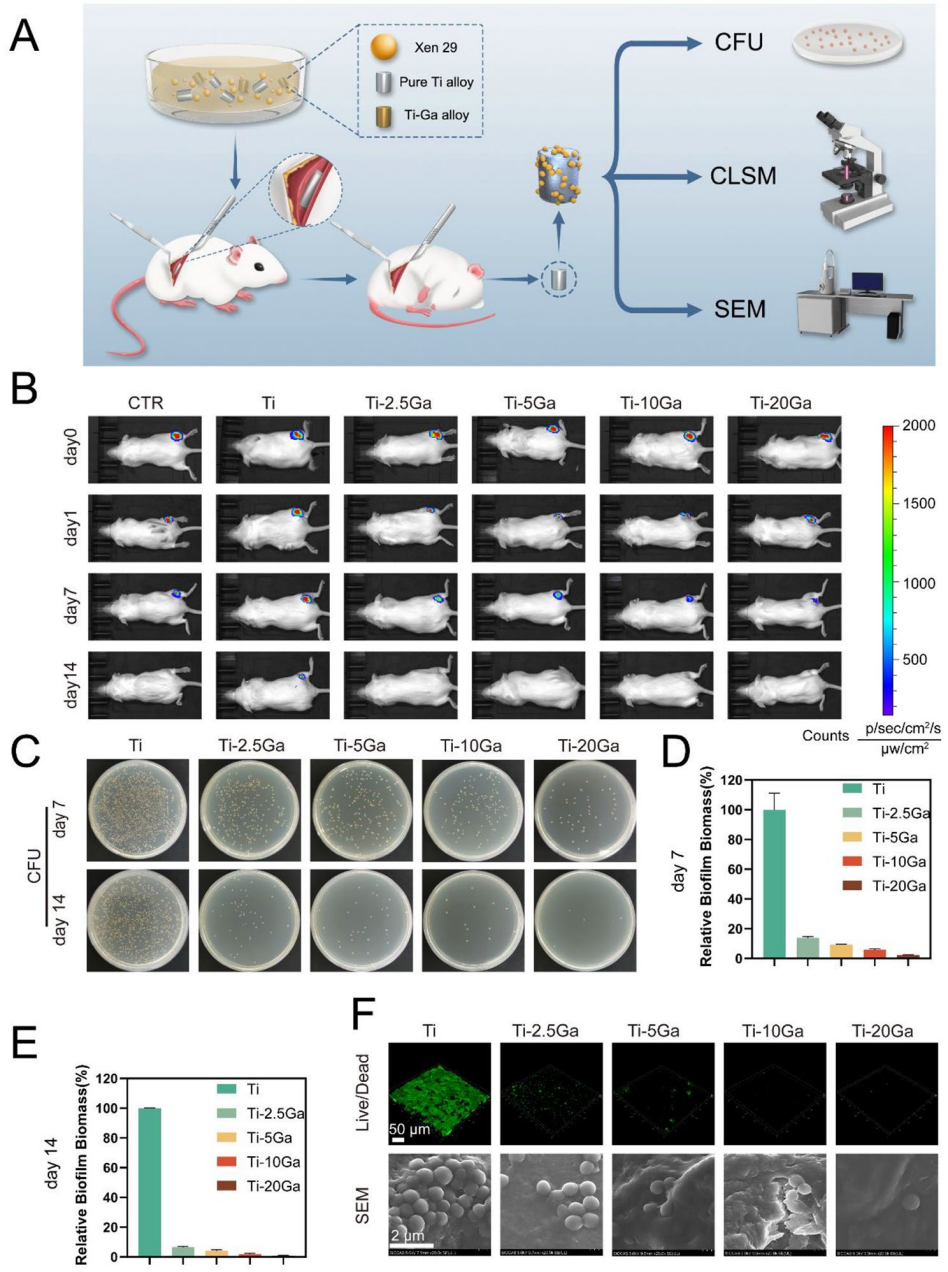
Systematic evaluation of the antibacterial effect of Ti-Ga alloys in vivo using the subcutaneous implant-related *S. aureus* infection model. **(A)** Schematic depicting the treatment regime. **(B)** Representative images of bioluminescence of the *S. aureus*-infected mice at different time points using an IVIS system. **(C)** Bacterial burden on the surface of alloys on days 7 and 14 was measured using the dilution coating plate method. **(D)** Quantitative analysis results of bacterial burden on day 7 of different groups. **(E)** Quantitative analysis results of bacterial burden on day 14 of different groups. **(F)** CLSM and SEM images of extracted alloys to characterize bacteria biofilm

A schematic illustration of the mouse implant infection model and various experiments of anti-biofilm infection evaluation are shown in Fig. 7A. Real-time bioluminescence monitoring was performed at four-time points to evaluate bacterial burden in all groups. As shown in Fig. 7B, on day 0, the mice in the six groups exhibited similar bioluminescence intensity, indicating a similar initial bacterial burden. The bioluminescence intensity in the Ti-Ga groups decreased dramatically on day 7, especially the Ti-20Ga group, while no bioluminescence was detected on day 14 in the Ti-Ga groups. The bacterial burden remained almost constant in the pure Ti group with a strong bioluminescence intensity, even on day 14, which was higher than that of the CTR group. We believe that biofilms formed on the Ti surface contribute to the pathogenesis of bacterial infections. The plate counting method was used to further quantify the bacterial load in mice following different treatments. The alloys from all groups were collected in a sterile manner on days 7 and 14. After removing the planktonic bacteria by washing with PBS, sessile bacteria embedded in the biofilm were obtained through sonication in an ultrasonic bath. The Ti-Ga alloy group demonstrated a strong anti-biofilm effect against *S. aureus* on days 7 and 14 by showing a noticeable decrease in bacterial CFU (Figs. 7 C-E). Confocal microscopy and SEM were used to visualize the biofilms on the surface of the alloys. As shown in Fig. 7F, a well-structured biofilm of dense bacteria formed on the surface of pure Ti after 7 days. The biofilm diminished gradually with increasing Ga content, which is consistent with the in vitro results. To sum up, the in vivo results demonstrated that Ti-Ga alloy had a good effect against biofilms.

### Histopathological evaluation

Furthermore, the inflammatory reaction of the subcutaneous muscular tissues was evaluated using H&E staining, and the bacterial residue in the peri-alloy soft tissues was assessed using Giemsa staining. As shown in Figure S15, large amounts of inflammatory cells infiltrated the peri-implant muscle tissues by day 7, except in the Ti-20Ga group. Among these, the pure Ti group exhibited the most inflammatory cell aggregation. Bacteria surrounding the alloy were further visualized using Giemsa staining (dark violet). Similarly, numerous bacteria were observed in the pure Ti group, whereas few bacteria were found in the Ti-20Ga group. These results indicated that the Ti-Ga alloy significantly inhibited biofilm-associated infection and reduced the bacterial load in tissues. On day 14, all groups demonstrated diminished inflammation except the pure Ti group (Figure S16). We hypothesize that chronic biofilm infections contributed to this. We observed that the harm caused by non-biofilm-associated infection was relatively low, and mice could even self-heal solely by relying on their immune system. However, once a biofilm forms, the infection becomes difficult to control [44]. Biofilms colonize pure titanium surfaces, acting as bacterial reservoirs, leading to intractable infections [45, 46]. Antibiotics fail to work for low metabolic rates of bacteria within the biofilm and another defense system of biofilm. It is the same for the body’s immune system.

### ***In vitro*** cytocompatibility of Ti-Ga alloys

The in vitro cytocompatibility was evaluated based on cellular activity after co-culturing the BMSC in the pure Ti and Ti-Ga alloy extracts for 1, 3 and 7 days (Figure S17). The cells co-cultured in the extract exhibited no obvious viability decrease, indicating that Ti-Ga alloy possessed satisfactory biocompatibility. The influence of Ga-based alloy extracts on the viability of BMSC cells was further verified using cytoskeletal staining (Figure S18). The cytoskeleton staining revealed that extracts of Ti-Ga alloys had no adverse effects on the spreading morphology of BMSC, which was consistent with the CCK-8 assays.

## Discussion

Biofilm-associated infections and aseptic osteolysis due to the overactivation of osteoclasts are two key factors that limit the clinical application of titanium-based implants. Our previous results showed that Ga^3+^ ions significantly inhibited biofilm formation and osteoclast differentiation, but the specific mechanism was unknown [20, 26]. It will be of great clinical significance to prepare a Ti-Ga alloy to simultaneously solve the above two problems. To this end, we prepared Ti-based alloys with different Ga content. XRD results proved that Ti alloys with Ga content of 2.5%, 5%, 10%, and 20% were successfully prepared. After the introduction of Ga, the Ga elements were uniformly distributed in the alloy. The above characteristics indicated that the Ti-Ga alloys had been successfully prepared and had potential biological applications.

Then we tested the anti-biofilm performance of Ti-Ga alloy in vivo and in vitro. Scanning electron microscopy, live-dead staining, colony counting, and crystal violet staining showed that *S. aureus* and *E. coli* formed a compact biofilm on the surface of pure Ti in vitro. In contrast, the biofilm structure was destroyed after the introduction of Ga^3+^ ions. In vivo results demonstrated that, compared with pure titanium, Ti-Ga alloy significantly inhibited subcutaneous implant-associated infection in mice, indicating that alloyed Ga still has excellent biofilm resistance performance and has the potential to be used as an orthopedic implant.

It was believed that Ga^3+^ ions play an antibacterial and anti-biofilm role by interfering with iron metabolism, but its specific mechanism is not clear. An in-depth understanding of the anti-biofilm mechanism of Ga^3+^ ions can further improve the antibacterial properties of Ti-Ga alloy. Therefore, we used proteomics to analyze the changes in the protein levels of *S. aureus* and *E. coli* after Ga^3+^ ions treatment. For *S. aureus*, the expression levels of proteins involved in iron uptake were significantly increased, including ferritic synthase and proteins involved in heme uptake, such as IsdB, lsdC, lsdl, etc., which are overexpressed when the bacterial sense an iron deficiency [47, 48]. Proteins responsible for Fe-S cluster assembly were significantly down-regulated after Ga^3+^ ions treatment, suggesting iron deficiency. In summary, the proteomics results proved that the iron metabolism of *S. aureus* was impaired after Ga^3+^ ions treatment, which further led to the disruption of the *S. aureus* biofilm. For *E. coli*, kegg’s results showed that Ga^3+^ ions treatment also led to iron metabolism disorders. We found that proteins involved in biofilm formation at the attachment stage were significantly enriched, especially proteins responsible for type I fimbriae, and other attachment-related proteins, including those involved in cellulose synthesis. Proteins involved in the synthesis and assembly of type I fimbriae, like fimbrial protein, pili assembly chaperone, N-terminal, and fimbrial biogenesis outer membrane usher protein decreased significantly [34, 35]. According to Wu, iron deficiency inhibited type 1 fimbriae synthesis by activating *fimE* [36]. We believed that Ga^3+^ ions might cause iron deficiency in *E. coli* and thus lead to adhesion organ dysfunction to inhibit biofilm formation. In vitro adhesion experiments demonstrated that the number of bacteria on the surface of Ti-Ga alloy decreased significantly after Ga^3+^ ions treatment, suggesting that Ga^3+^ ions can indeed interfere with the adhesion stage of *E. coli* biofilm formation.

In this work, we also explored the performance and mechanism of Ti-Ga alloys inhibiting osteoclast differentiation. TRAP staining, F-actin ring staining, SEM observation for bovine bone slices, and qRT-PCR showed that low-content Ga alloy could inhibit the differentiation and function of BMMs, which was consistent with previous reports. It’s worth noting that the specific mechanism by which Ga^3+^ ions inhibited osteoclasts is unclear. We found that the iron content in BMMs decreased after Ga^3+^ ions treatment detected by the iron probe. According to Zhang, hepcidin can inhibit osteoclast differentiation by reducing intracellular iron content, and we believe that Ga^3+^ ions inhibited osteoclast differentiation by a similar mechanism [40]. As a Fenton catalyzer, iron can accelerate the production of reactive oxygen species, an important signal of osteoclast activation [41]. We verified this by a ROS probe. After the addition of RANKL, intracellular ROS content in BMMs increased, while after Ga^3+^ ions treatment, intracellular ROS content almost returned to an average level, which was consistent with our hypothesis. TRAP staining, F-actin ring staining, and SEM observation for bovine bone slices also showed that supplementing iron to BMMs based on Ga^3+^ ions treatment could reverse the inhibition of osteoclast differentiation. Finally, the results of WB and immunofluorescence showed that Ga^3+^ ions inhibited the NF-κB pathway. In conclusion, Ga^3+^ ions may inhibit osteoclast differentiation through the iron-ROS -NF-κB axis.

## Conclusion

A series of Ga-doped Ti alloys were synthesized and their anti-biofilm mechanism and effects against *S. aureus* and *E. coli* and inhibiting RANKL-dependent osteoclast maturation mechanism were verified in this study. We identified that Ga^3+^ ions could bring chaos to iron metabolism and affect biofilm formation. And the anti-biofilm effect of Ti-Ga alloys showed a strong inhibitory effect on biofilm formation in a Ga content-dependent manner both in vitro and in vivo. In addition, the Ti-Ga alloy displayed apparent inhibitory effects on osteoclast differentiation, which contributes to osteolysis in orthopedics. Western blot results further revealed that Ga^3+^ ions inhibited the activation of the NF-κB pathway by affecting iron metabolism and prevented P65 from entering the osteoclast nucleus, thus downregulating the transcription of osteoclast-specific genes. Taken together, this study suggests that the Ti-Ga alloy possessed considerable biocompatibility, antibiofilm, and inhibiting osteoclast differentiation activity, permitting their potential use as a promising orthopedic implant material.

## Electronic supplementary material

Below is the link to the electronic supplementary material.

Supplementary Material 1

Supplementary Material 2

## Data Availability

The datasets used and/or analyzed during the current study are available from the corresponding author on reasonable request.

## Abbreviations

Ga: Gallium
M-CSF: Macrophage colony-stimulating factor
RANKL: Receptor activator of nuclear factor-κB ligand
XRD: X-rays diffraction
TEM: Transmission electron microscope
APT: Atom probe tomography
BMSC: Bone mesenchymal stem cells
BMMs: bone marrow-derived macrophages
FBS: Fetal bovine serum
OD: Optical density
PBS: Phosphate-buffered saline
SDS-PAGE: SDS sulfate-polyacrylamide gel electrophoresis
FITC: Fluorescein isothiocyanate
SPF: Specific-pathogen-free
DEPs: Differentially expressed proteins
CTR: Control
