# Supplementary material for: A dual functional Ti-Ga alloy: inhibiting biofilm formation and osteoclastogenesis differentiation via disturbing iron metabolism
Source: Biomater Res. 2023 Mar 29;27:24. doi: 10.1186/s40824-023-00362-1 (PMC10053110; doi:10.1186/s40824-023-00362-1)
Supplement: Supplementary file 1 — Supplementary Material 1 [file 40824_2023_362_MOESM1_ESM.docx]

**Supporting Information**

**A Dual Functional Ti-Ga Alloy: Inhibiting Biofilm Formation and Osteoclastogenesis Differentiation via Disturbing Iron Metabolism**

Fupeng Li^1†^, Kai Huang^1†*^, Jinbing Wang^2†^, Kai Yuan^1^, Yiqi Yang^1^, Yihao Liu^1^, Xianhao Zhou^1^, Keyu Kong^1^, Tao Yang^3^, Jian He^4^, Chunjie Liu^4^, Haiyong Ao^5^, Fengxiang Liu^1*^, Qian Liu^6*^, Tingting Tang^1*^, Shengbing Yang^1*^

^1^ Department of Orthopaedic Surgery, Shanghai Ninth People’s Hospital, Shanghai Jiao Tong University School of Medicine, Shanghai Key Laboratory of Orthopaedic Implants, Shanghai, 200011, China

^2^ Department of Oral and Maxillofacial-Head and Neck Oncology，Shanghai Ninth People's Hospital, Shanghai Jiao Tong University School of Medicine, College of Stomatology, Shanghai Jiao Tong University, National Center for Stomatology, National Clinical Research Center for Oral Diseases, Shanghai Key Laboratory of Stomatology, Shanghai Research Institute of Stomatology,

Shanghai, 200011, China

^3^ Department of Materials Science and Engineering, Hong Kong Institute for Advanced Study,

College of Science and Engineering, City University of Hong Kong, Hong Kong, China

^4^ M-Duke Medical Technology (Shanghai) Co., Ltd, Shanghai, China

^5^ Jiangxi Key Laboratory of Nanobiomaterials & School of Materials Science and Engineering,

East China Jiaotong University, Nanchang, 330000, China

^6^ Department of laboratory medicine, Ren Ji Hospital, Shanghai Jiao tong university school of medicine, Shanghai, 200127, China

^†^These authors contributed equally to this work.

* Corresponding Authors.

E-mail addresses: K. Huang ([kai_huang@aliyun.com](mailto:kai_huang@aliyun.com)), F. Liu ([liu_fengxiang@126.com](mailto:liu_fengxiang@126.com)), Q. Liu ([liuqian_rj@shsmu.edu.cn](mailto:liuqian_rj@shsmu.edu.cn)), T. Tang ([ttt@sjtu.edu.cn](mailto:ttt@sjtu.edu.cn)), S. Yang ([shengbingyang@shsmu.edu.cn](mailto:shengbingyang@shsmu.edu.cn)).


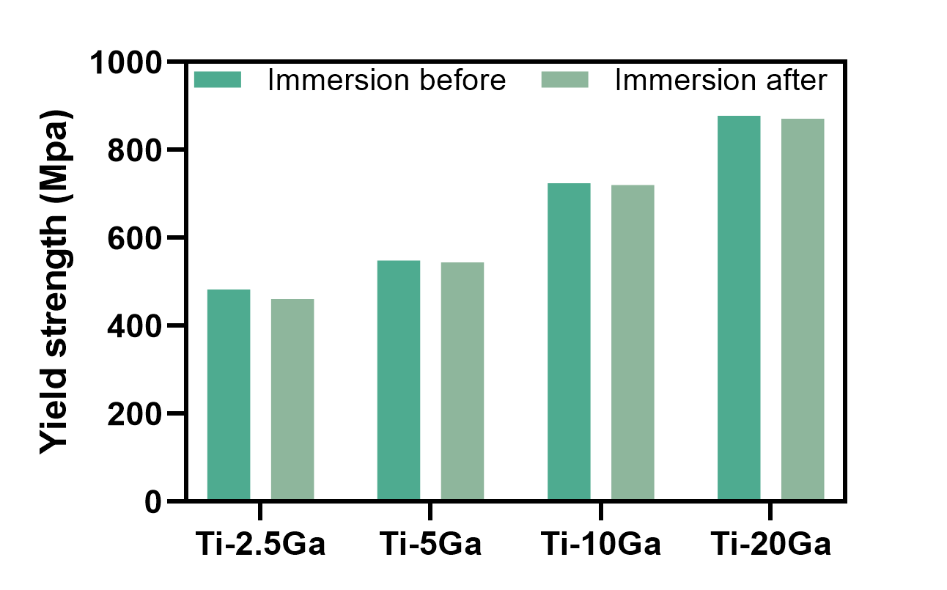


**Figure S1.** Yield strength of Ti-Ga alloys.

**Figure S2.** Stress-strain curve of Ti-Ga alloys.


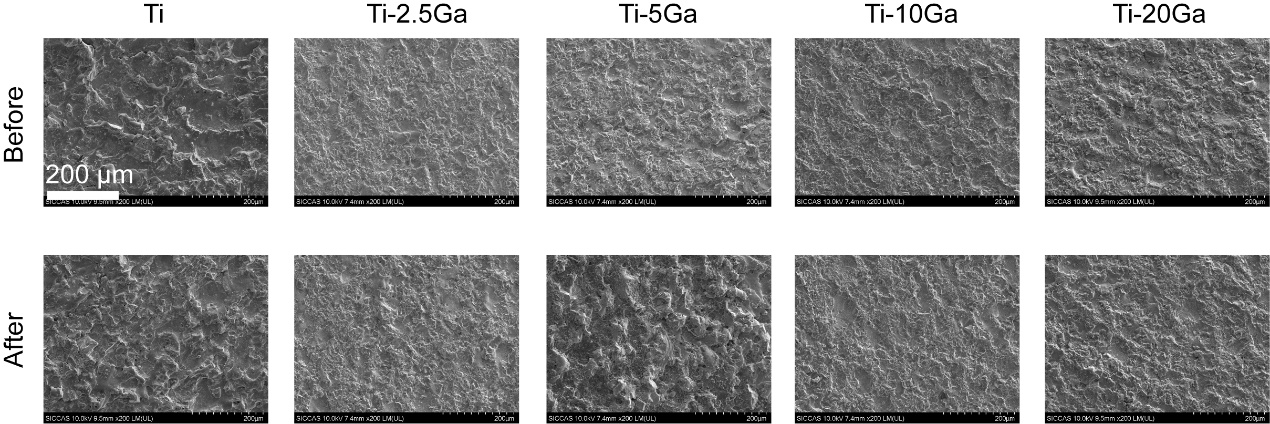


**Figure S3.** Corrosion morphology after 30 days’ immersion in SBF solution.





**Figure S4.** The releasing profile of Ga ion.


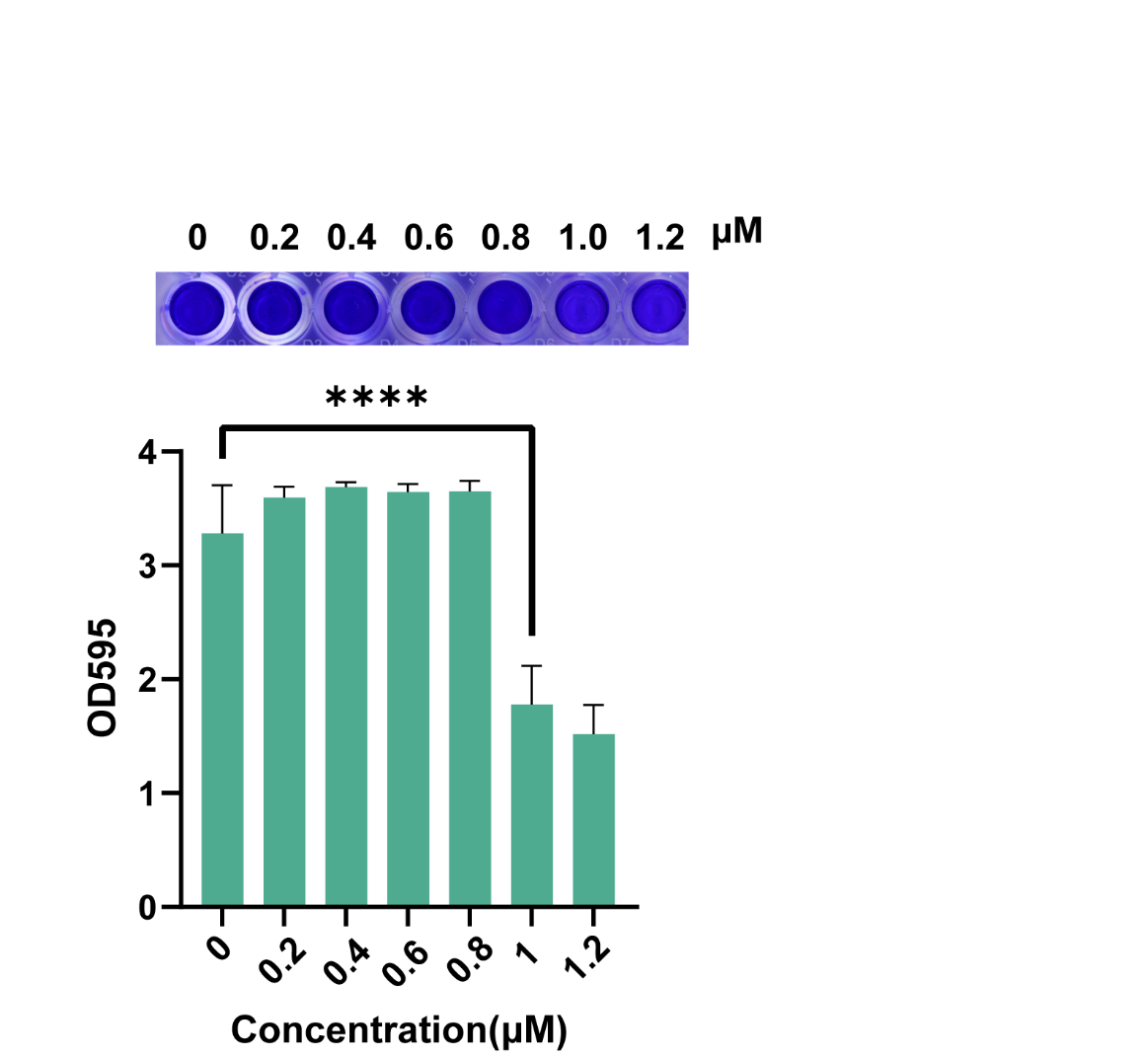


**Figure S5.** Minimum biofilm inhibitory concentration (MBIC) testing of gallium ion against *S. aureus*. Biofilms were stained with crystal violet.


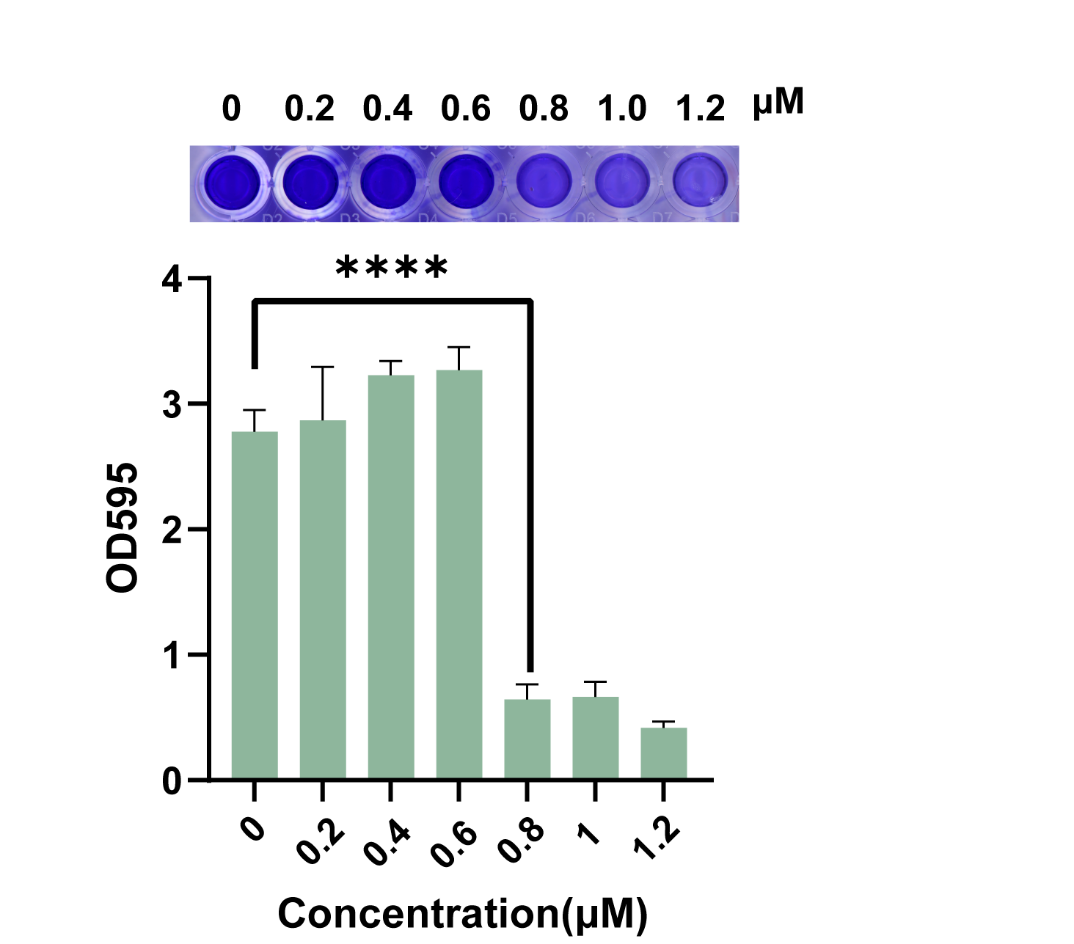


**Figure S6.** Minimum biofilm inhibitory concentration (MBIC) testing of gallium ion against *E. coli*. Biofilms were stained with crystal violet.


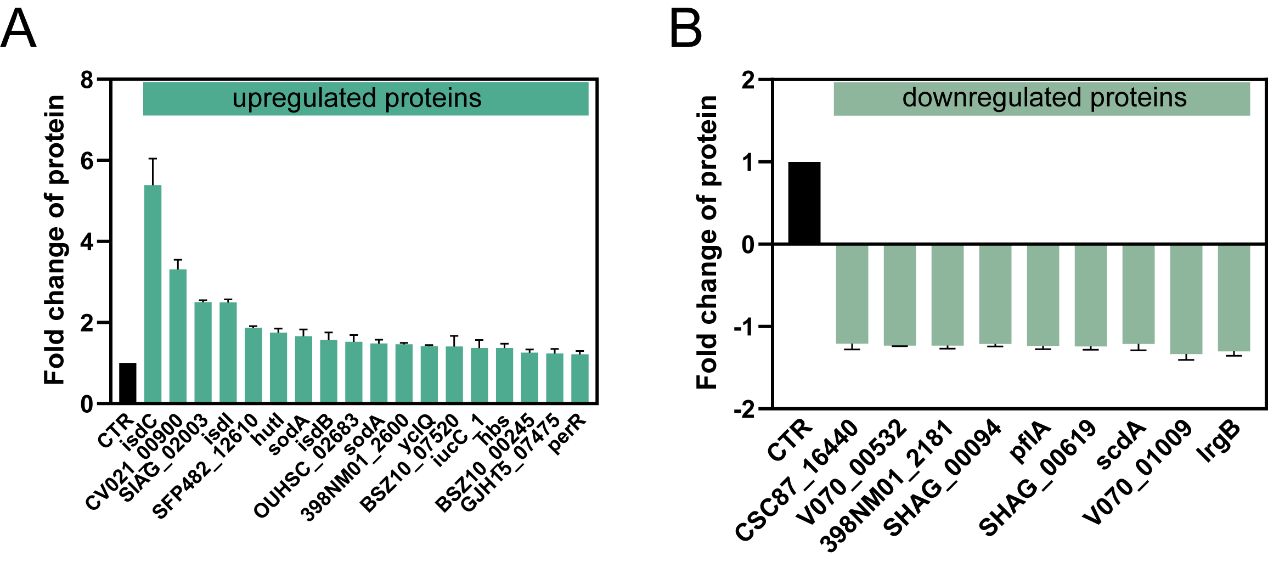


**Figure S7.** Quantitative analysis of iron metabolism related proteins of *S. aureus*.


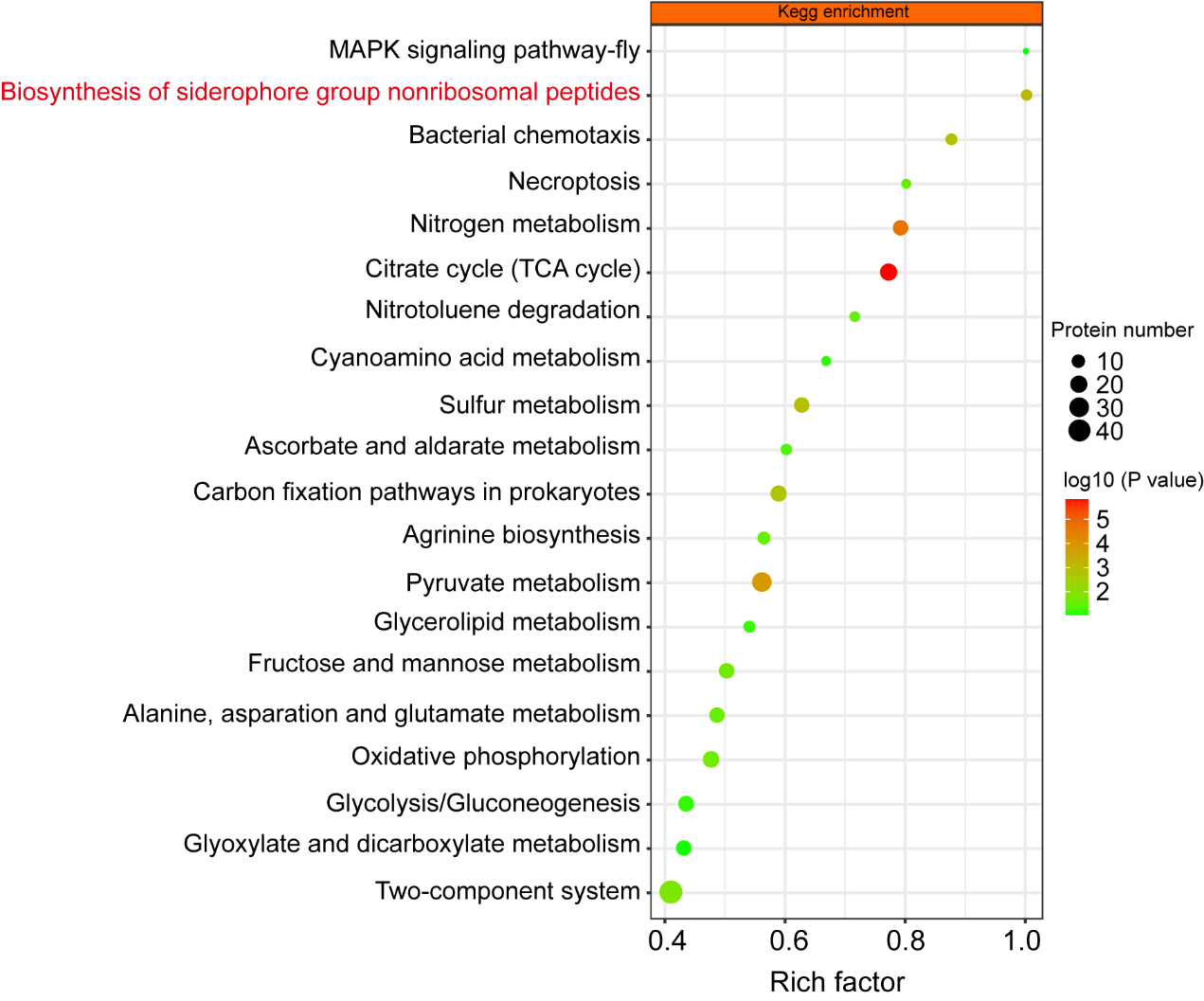


**Figure S8.** DEPs enriched in the KEGG pathway.

**
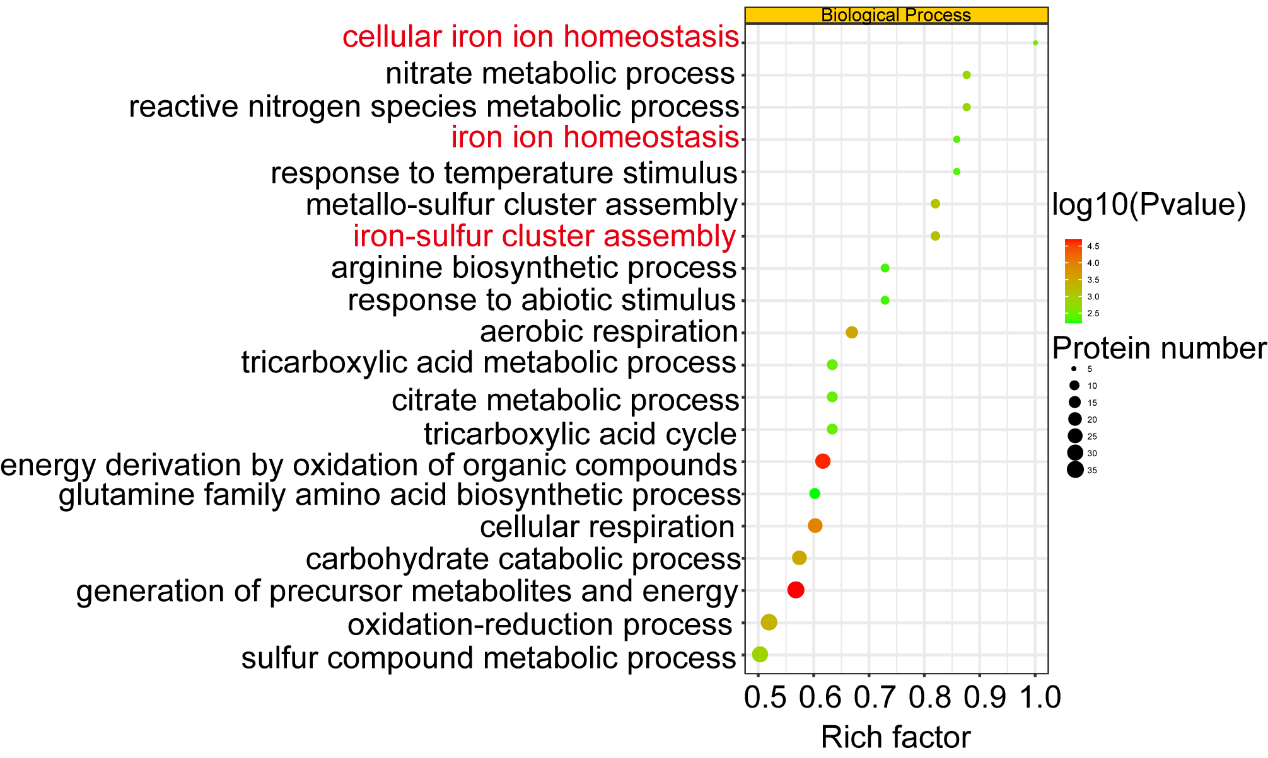
**

**Figure S9.** GO enrichment analysis (Biological process) of differentially expressed proteins after gallium treatment.

**
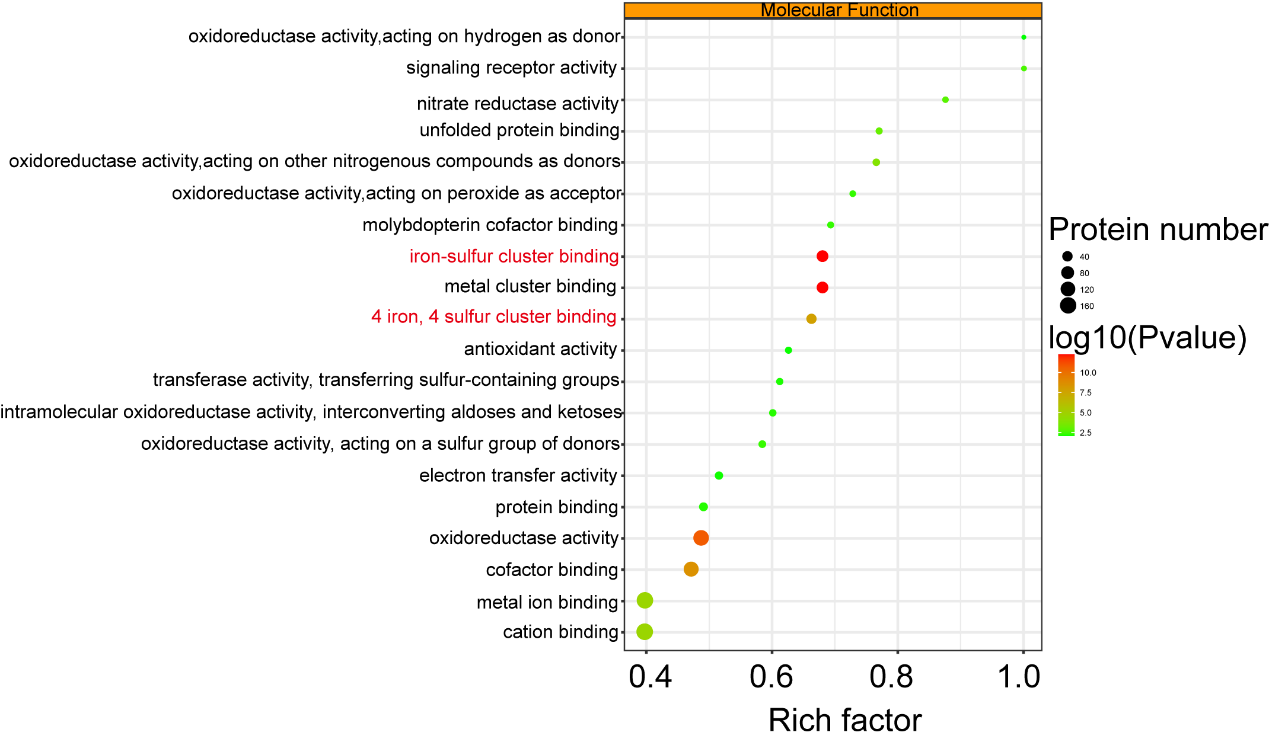
**

**Figure S10.** GO enrichment analysis (Molecular function) of differentially expressed proteins after gallium treatment. **
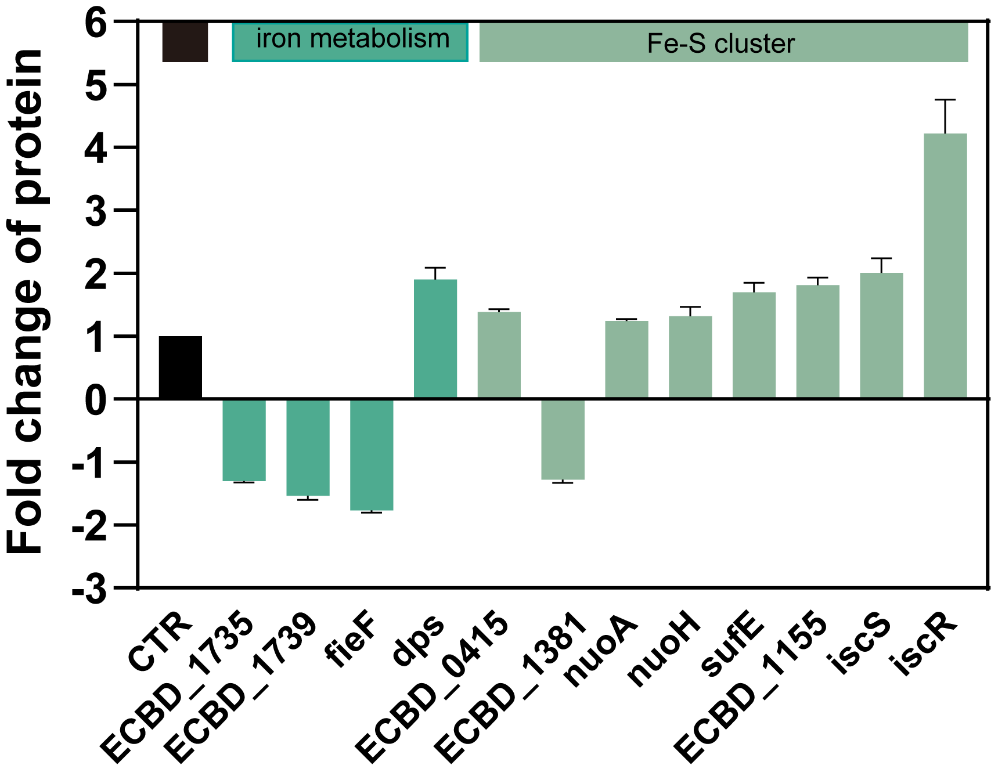
**

**Figure S11.** Quantitative analysis of upregulated and downregulated iron metabolism related proteins of *E. coli* after gallium treatment.


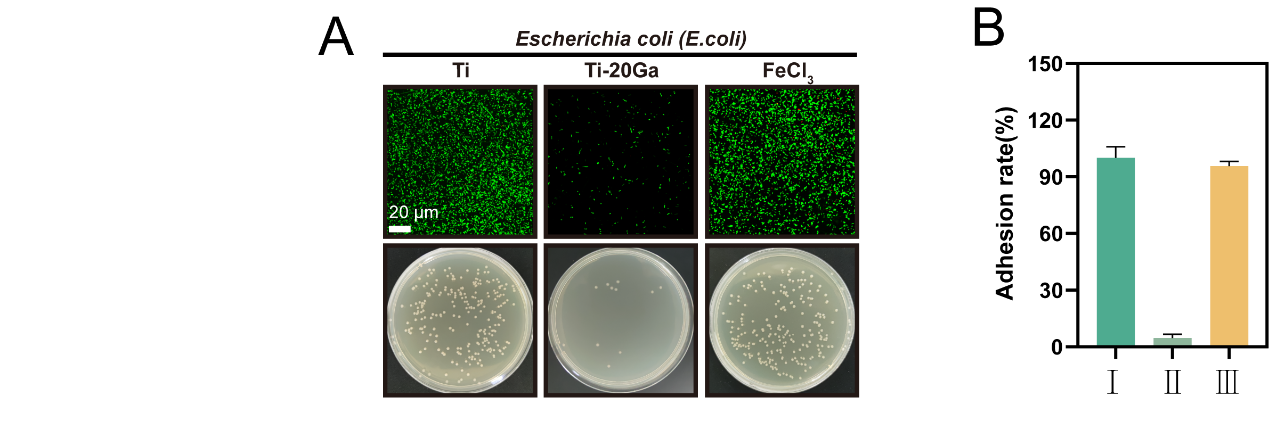


**Figure S12. A)** Adherence assay through confocal microscopy and CFU formation. **B)** Quantification of CFU formation (Ⅰ:Ti, Ⅱ:Ti-20Ga, Ⅲ: Ti-20Ga+FeCl_3_).

**
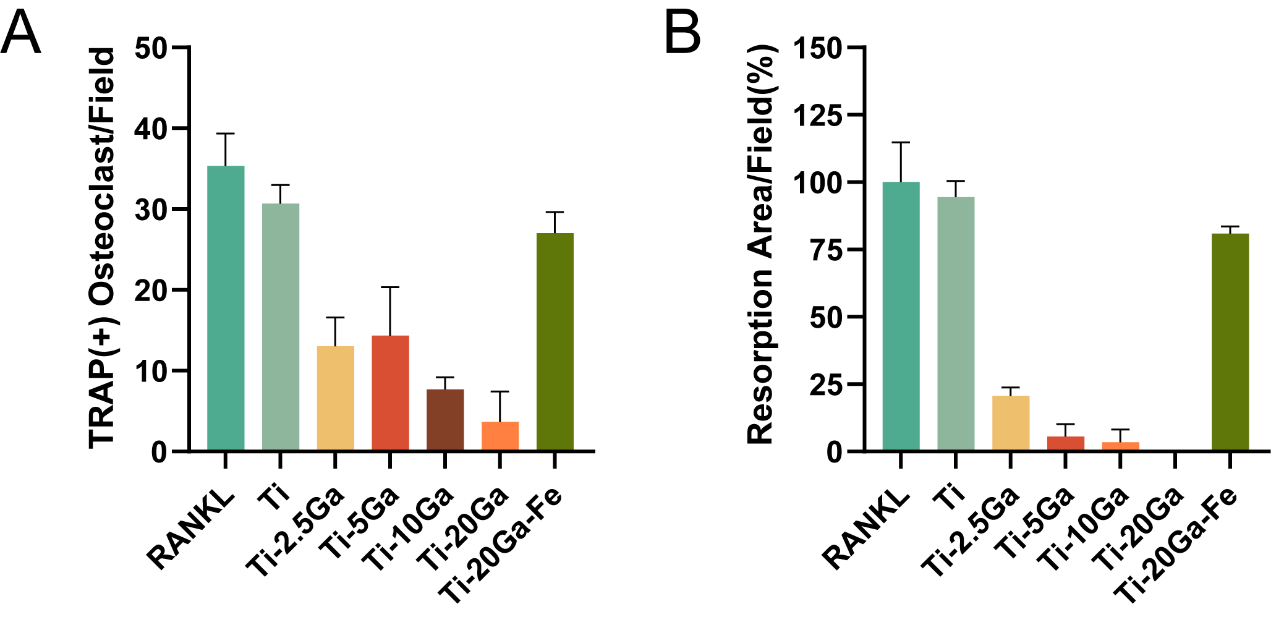
**

**Figure S13.** Quantification of TRAP-positive multinucleated osteoclasts that were treated with different Ti-Ga alloy extracts. (50 ng mL^-1^, RANKL).

**
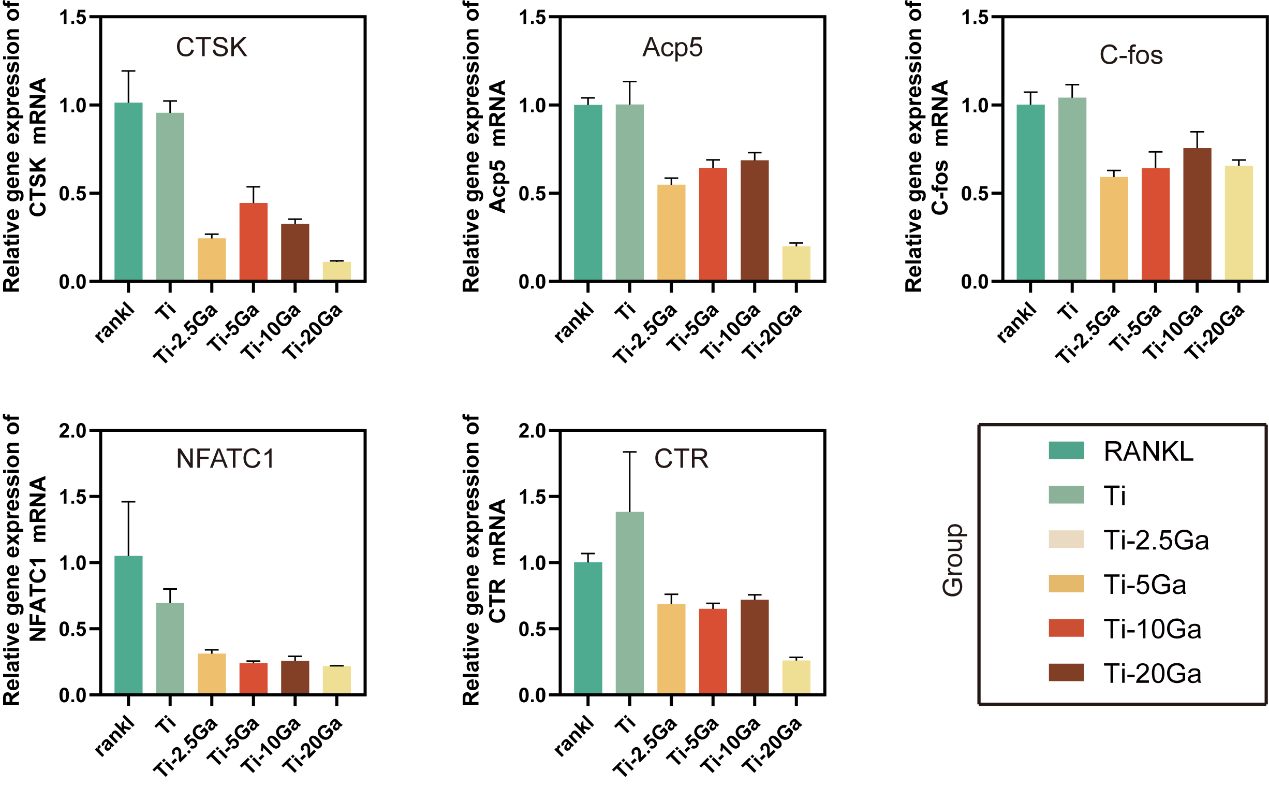
 Figure S14.** qPCR analysis of expression of the osteoclast-specific genes *Acp5*, *C-fos*, *CTR*, *Ctsk*, and *NFATC1* relative to *β-actin* in BMMs that were stimulated with RANKL in the presence of extracts of Ti and Ti-Ga alloy.


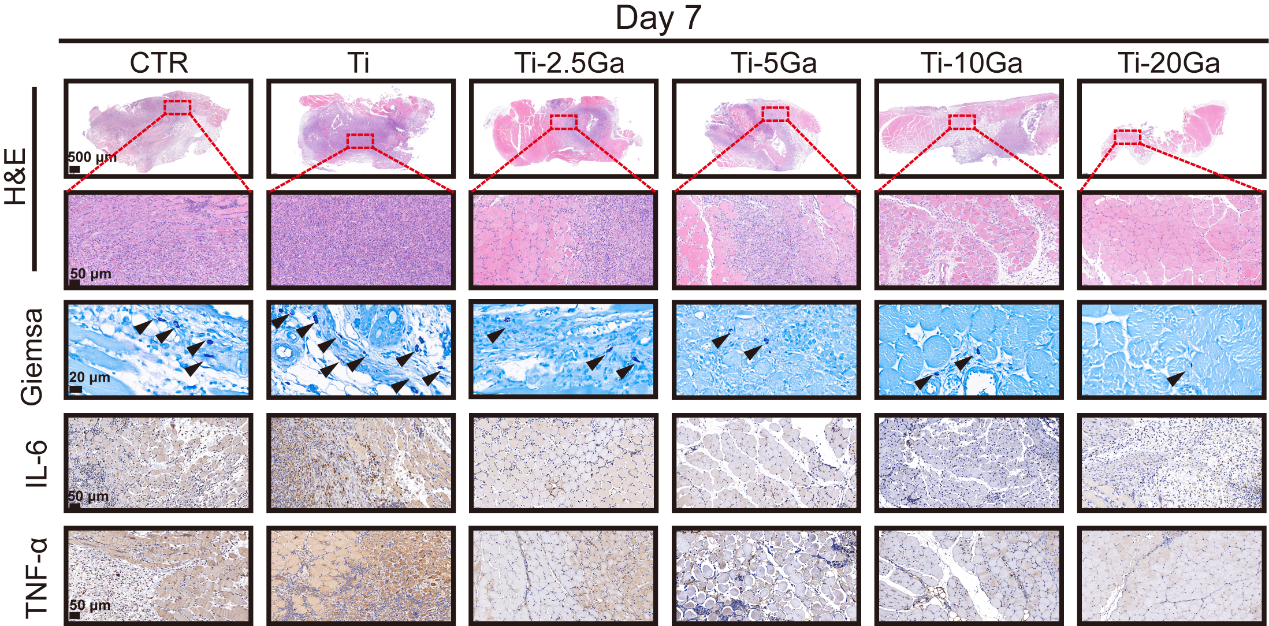


**Figure S15.** Hematoxylin and eosin (H&E) staining and Giemsa staining of the bacterial biofilm infected muscle tissues after different treatments on day 7.


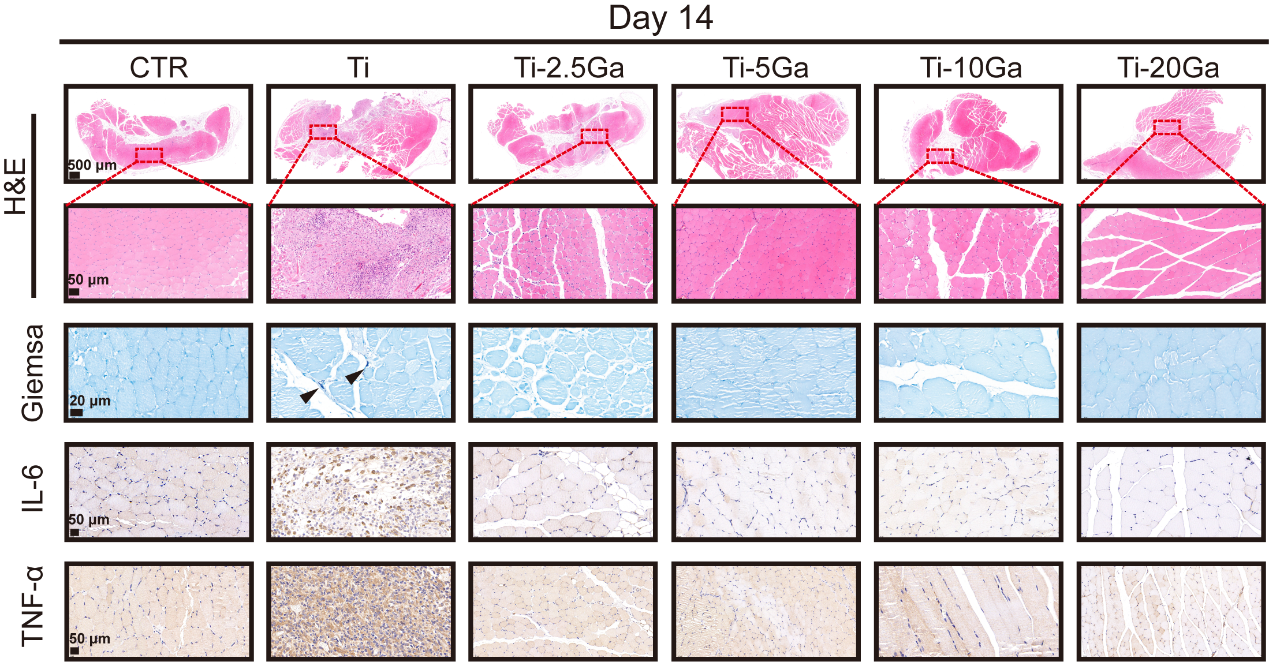


**Figure S16.** H&E staining and Giemsa staining of corresponding infected soft tissues on day 14.

**Figure S17.** Viability of L929 cells treated with extracts of Ti and Ti-Ga alloy.


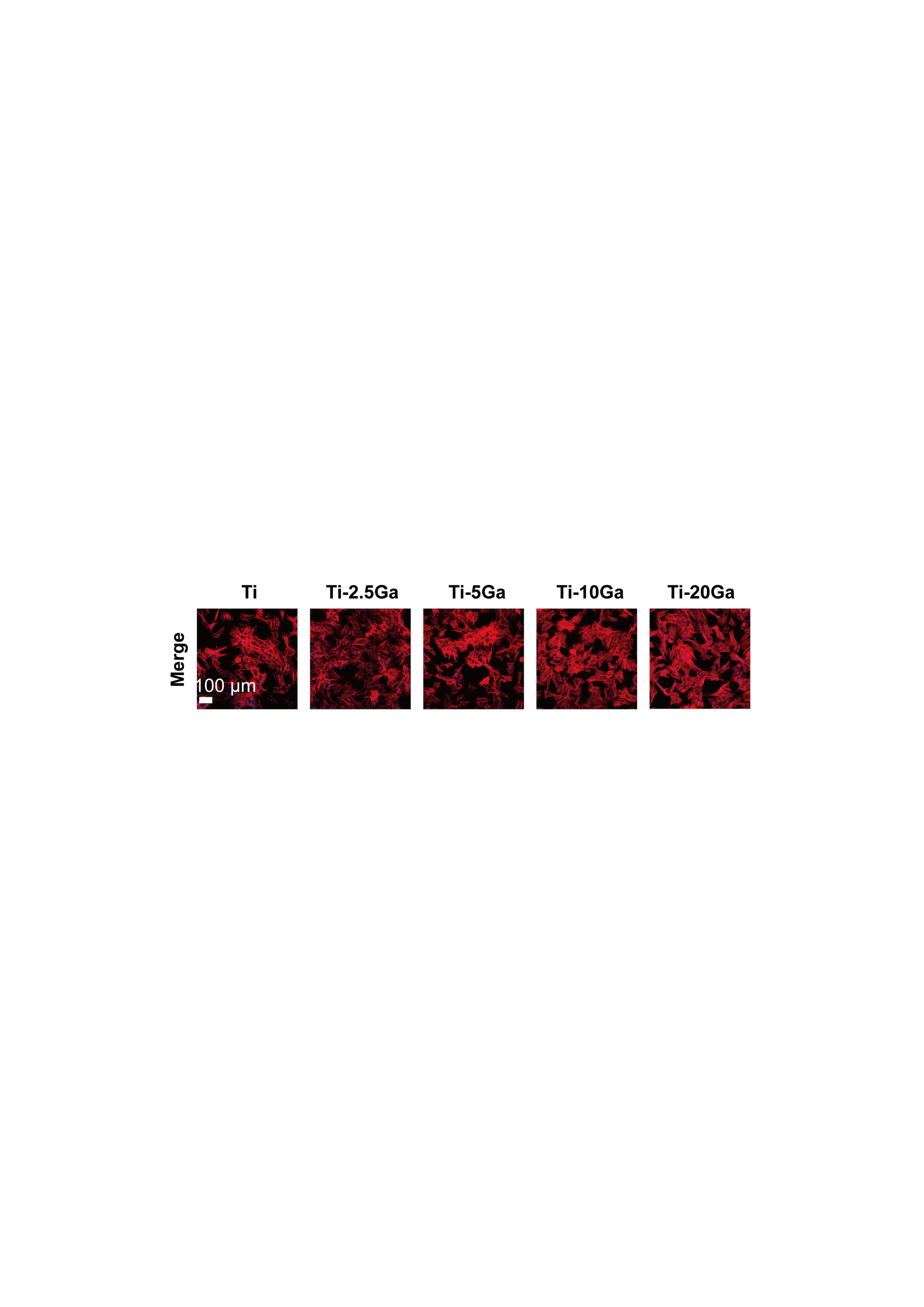


**Figure S18.** Cytoskeletal staining for BMSC cells cultured with extracts of Ti and Ti-

Ga alloy extracts.
