# Supplementary material for: A dual functional Ti-Ga alloy: inhibiting biofilm formation and osteoclastogenesis differentiation via disturbing iron metabolism
Source: Biomater Res. 2023 Mar 29;27:24. doi: 10.1186/s40824-023-00362-1 (PMC10053110; doi:10.1186/s40824-023-00362-1)
Supplement: Supplementary file 2 — Supplementary Material 2 [file 40824_2023_362_MOESM2_ESM.docx]

February 21, 2023

Editor-in-Chief Dr. Byeongmoon Jeong,

*Biomater. Res.*

Dear Editors and Reviewers,

Thank you very much for taking the time to review our manuscript (Research Article, BMRS-D-22-00305). We greatly appreciate all the comments and suggestions! Based on the instructions provided in your letter, we uploaded the file of the revised manuscript and a copy with all the changes highlighted in yellow.

Our point-by-point responses to the comments are appended to this letter. The comments are reproduced, and our responses are provided directly afterward.

Thank you for your consideration. I look forward to hearing from you.

Sincerely,

Shengbing Yang, Ph.D.

Associated professor, Shanghai Key Laboratory of Orthopaedic Implants

Shanghai Ninth People’s Hospital, Shanghai Jiao Tong University School of Medicine

No.639 Zhizaoju Road

Shanghai, China 200011

E-mail: shengbingyang@shsmu.edu.cn

Reviewer #1: This paper describes the preparation of Ti-Ga alloy and its effects on inhibiting biofilm formation and osteoclastogenesis differentiation. As described in the manuscript, authors' previous work (refs. 20 & 23) already demonstrated that the Ga^3+^ ion containing materials can have such effects. However, the detailed/reasonable mechanism have not been clear. The novelty of this work is to examine how Ga^3+^ ions can have antibacterial effects and inhibit osteoclast differentiation. While the claims in this study are strongly supported by the experimental data, the following points should be addressed before publication.

1. Author explained that "the Ti-Ga alloy-induced bacterial adhesion inhibition resulted from the Ga^3+^ ions release from the alloy". How much of Ga^3+^ ions are released? What would be the minimally required concentration of Ga^3+^ ions for such antibacterial effect?

Response:

Thank you for your valuable suggestions. The releasing profile of Ga ion has been added. In addition, the crystal violet stain experiment has been performed to determine the minimally required concentration of Ga^3+^ ions for the anti-biofilm effect. Gallium ion at 1 μM could reduce *Staphylococcus aureus* (*S.aureus*) adhesion and prevent biofilm formation. At the same time, the MBIC of the Ga ion for *Escherichia coli* (*E. coli*) was 0.8 μM. In a word, the released gallium from Ti-Ga alloy could inhibit biofilm formation.

**Figure S4.** The releasing profile of Ga ion.

**Figure S5.** Minimum biofilm inhibitory concentration (MBIC) testing of gallium ion against *S. aureus*. Biofilms were stained with crystal violet.

**Figure S6.** Minimum biofilm inhibitory concentration (MBIC) testing of gallium ion against *E. coli*. Biofilms were stained with crystal violet.

2. Regarding in vitro anti-adherence assays, Ti-Ga alloys were incubated in bacterial suspension for 2 h. What if the alloy would be incubated in *E. coli* (or *S. aureus*) suspension for longer time such as 24 h?

Response:

Thank you for your comments. Biofilm formation is a finely regulated multi-stage orderly process, including bacterial adhesion, biofilm formation, biofilm maturation, and biofilm dispersal [1]. In the early stage, such as 2 hours, bacteria adhere to the surface of the alloy [2, 3]. As for 24 hours, biofilm has already formed after 24 hours [4, 5]. Therefore, we chose the early 2 hours for the adhesion experiment.

Reviewer #2: This manuscript describes dual function of Ti-Ga alloy materials for biofilm inhibition and osteoclastogenesis. The authors carefully studied the biological responses on materials surface including antibacterial effect and osteoclast differentiation. Various in vitro tests and in vivo experiments were well performed and characterized. However, there are several concerns on the effect of Ga ion for osteogensis and osseointegration. This reviewer suggests major revision of the manuscript before consideration for the publication.

Comments

1. The authors mainly focused on osteoclastogenesis by Ti-Ga alloy. However, osteogenesis of implanting material surface is also an important factor for successful osseointegration. The authors only tested osteocalstogenesis but this reviewer suggests characterization of osteoblast viability, proliferation, and osteogenic differentiations, in the presence of Ti-Ga alloys.

Response:

Thank you for your instructive suggestions. The effect of alloy surfaces on osteogenesis is a concern that needs to be carefully studied. However, osteolysis caused by the long-term use of titanium alloy is mainly caused by the overactivation of osteoclasts, which is also the main problem this paper intends to focus on. Our results indicated that the titanium gallium alloy could effectively inhibit osteoclast differentiation. Your comments are extremely instructive, scientifically valuable, and align with our thoughts. We have studied in detail the influence of Ti-Ga alloy on bone integration in another paper, which is being submitted to *Composite Part B: Engineering*, and here we show some contents you concern about, and the conclusion is that the Ti-Ga could improve osseointegration.

**Figure R1.** Proliferation test of BMSCs cocultured with Ti and Ti–Ga alloy extracts.

**Figure R2.** Differentiation of BMSCs cultured in Ti and Ti–Ga alloy extracts. ALP-stained BMSCs cultured in Ti and Ti–Ga alloy extracts for 7 days A) and 14 days B) after osteogenic differentiation stimulation. C) Corresponding ALP activity of the BMSCs. D) Alizarin red-stained BMSCs after culturing for 21 days. E) Relative expression of osteogenic differentiation marker genes in BMSCs cultured for the indicated times in an osteogenic medium with pure Ti and Ti–Ga alloys extracts, given as mean ± standard deviation (**p* < 0.05; ***p* < 0.01).

2. A quantitative analysis of Ga ion releasing (releasing profile) in a physiological condition should be monitored at least during the osseointegration period (4 - 8 weeks).

Response:

Thank you for your instructive suggestions. A quantitative analysis of Ga ion releasing (releasing profile) in a physiological condition has been monitored within 8 weeks according to your requirement.

**Figure S4.** The releasing profile of Ga ion.

3. What is exact meaning of Ti-Ga alloy extract in this work? Some of characterizations were performed directly on the Ti-Ga alloy substrates but several tests regarding cellular response were done on wellplate and then applied alloy extract. The experimental method is not clear. If the authors tested using Ga ion rather than Ti-Ga alloy disc, it should be explained. This reviewer suggests observation of all the cell-related experiments on Ti-Ga alloy disc rather than using or adding extract substances to see the exact cellular response on the surface.

Response:

Thank you for your comment. As far as we know, the coculture of cells with alloy extracts and direct co-culture of cells with alloy are equivalent. For example, Qu *et.al* used Zn-Ag alloy extracts to study the effect of Zn-Ag alloy on osteoclast differentiation, and Zn-Cu alloy extract to study the effect of Zn-Cu alloy on osteoblast differentiation [6-9].

4. One of major concern is mechanical properties of Ti-Ga alloy after Ga ion releasing. Also, is there any surface morphology or structural change after Ga ion releasing from the alloy? The mechanical properties (in the period of osseointegration, usually 4-8 weeks) need to be studied together with Ga ion releasing properties as commented in comment #2.

Response:

Thank you for your valuable suggestions. The mechanical properties of Ti-Ga alloys in the period of osseointegration (8 weeks) are presented in **Figure S1-S2**. With the increase of gallium content, the yield strength (YS) of Ti-Ga alloy increases obviously, indicating that gallium improved the mechanical property of pure Ti. When the content of gallium reaches 20%, the YS of Ti-Ga alloy reaches 877.93MPa, which is much higher than that of pure Ti (240-550 MPa) [10, 11]. In addition, after 8 weeks of immersion, the mechanical properties of Ti-Ga alloys have almost no change. The YS of Ti-20Ga only decreased from 877.9MPa to 870.8Pa. We also explored the surface morphology of alloys after immersion and the results are as follows. No apparent changes were observed **Figure S3**.

**Figure S1.** Tensile strength of Ti-Ga alloys.

**Figure S2.** Stress-strain curve of Ti-Ga alloys.

**Figure S3.** Corrosion morphology after 30 days’ immersion in SBF solution.

5. In figure 5B, the fluorescence intensity of RANKL+5Ga group shows higher intensity comparable to controls. If possible, fluorescence intensity comparison (as a bar graph) needs to be suggested.

Response:

Thank you for your valuable suggestions. Immunofluorescence staining was used to evaluate osteoclast differentiation further, and mature osteoclasts are multinucleated giant cells with a larger cytoskeleton. We stained the cytoskeleton with ghost-pen cyclic peptide, and the red ring fluoresces as an F-ACTIN ring. The size and number of rings were indicators of osteoclast differentiation, and were independent of fluorescence intensity. Hence, we believe it's not necessary to compare the fluorescence intensities.

6. In figure S11, inflammatory regions are observable in Ti-Ga alloy samples outside of red boxes regions. Authors should suggest and explain based on the whole tissue analysis.

Response:

Thanks for your comment. In the subcutaneous implant infectious model, we collected the muscle tissue around the implant for sectioning and staining. The red box in the figure selects a typical visual field for magnification. The inflammatory trend reflected in the typical visual field, which is the same as in the low-magnification visual field.

7. In vitro cytocompatibility test in figure S13, the cell name is L929 while in the manuscript, it is BMSC. Also, why there is no proliferation of cells from 1 day to 7 days in Figure S13? What is control of cell viability test in this work? The authors should suggest proliferation test for this kind of cellular behavior monitoring.

Response:

Thank you for your reminder. BMSCs in the manuscript were written by mistake, and the cells we used to perform the cytocompatibility test were L929 which was regularly used to evaluate the cytotoxicity of biomaterials [12, 13]. In this experiment, we assessed the effect of alloys’ extracts on L929 activity from 1 to 7 days by comparing with the control group’s OD value of each day. As you had pointed out, this could not reflect the cell proliferation. According to your suggestion, we changed the calculation method, setting day 1 control group as the base line to observe the proliferation change, and the result had been listed as below. The result indicated that Ti-Ga alloys would not affect the cells proliferation.

**Figure S17.** Viability of L929 cells after different treatment.

**Figure S17.** Viability of L929 cells after different treatments.

8. In Figure 5a, what is white circles in the upper-left region?

Response:

Thank you for your comment. The TRAP experiment was carried out on a 96-well plate as described in Materials and Methods. After dyeing, we took photos of each well and chose the typical field to magnify. To fully display the situation in the whole hole, we fully displayed each hole and its surroundings. The white circles you mentioned are the wholes of 96 wells.

9. In the figure, the letter size in Figure 3B, 4B, and 4C should be increased. It is hard to recognize.

Response:

Thanks for your reminder. The letter size has been increased for better recognition.

 **Figure 3.** Proteomic revealed iron disorder of *S. aureus* after Ga(Ⅲ) treatment. A) Volcant plot of *S. aureus*. B) Kegg enrichment of *S. aureus*. C) Heat map of hierarchical clustering analysis of the iron metabolism-related proteins after Ga treatment. D) Possible mechanism of Ga disrupting *S. aureus* iron metabolism.

 **Figure 4.** Decreased attachment capacity of *E. coli* meditated by iron deficiency after Ga treatment. A) Volcant plot of *E. coli*. B) GO enrichment analysis (Cellular component) of differentially expressed proteins after Ga treatment. C) Heat map of hierarchical clustering analysis of the differentially expressed proteins after Ga treatment. D) Quantitative analysis of type 1 fimbriae assembly related proteins of *E. coli*) The possible mechanism of Ga disrupting *E. coli* biofilm formation at the adhesion stage.

10. Is the Ti-Ga20 optimized condition? What would be the problem when the Ga composition increase more? It needs to be addressed in the manuscript.

Response:

Thanks for your comments. In this manuscript, we synthesized a series of Ti-Ga alloys. Among them, Ti-20Ga displayed the best anti-biofilm effect. According to the mechanical property test results, with the elevation of gallium content, the mechanical properties of Ti-gallium alloy are gradually enhanced, especially the yield strength. It is observed that the yield strength of Ti-Ga alloy is 877.9 Mpa when the content of gallium is 20%. This is close to or even exceeds the yield strength of conventional Ti6Al4V alloys [14, 15]. The excessively strong mechanical properties are not conducive to application *in vivo*. Considering that titanium alloy has excellent anti-biofilm and anti-osteolysis effects when the content of gallium is 20%, we believe that it is not necessary to explore the situation when the content of gallium is more than 20%.

**Reference:**

[1] C.R. Arciola, D. Campoccia, L. Montanaro, Implant infections: adhesion, biofilm formation and immune evasion, Nat. Rev. Microbiol. 16(7) (2018) 397-409.

[2] N.A.a.A.U.K. Azna Zuberi, crisPri induced suppression of Fimbriae gene (fimH) of a Uropathogenic Escherichia coli: an approach to inhibit Microbial Biofilms, Front. Immunol. 8 (2017) 1-8.

[3] L.M. Marin, Y. Xiao, J.A. Cury, W.L. Siqueira, Modulation of Streptococcus mutans Adherence to Hydroxyapatite by Engineered Salivary Peptides, Microorganisms 10(2) (2022) 223-235.

[4] G. Guo, H. Zhang, H. Shen, C. Zhu, R. He, J. Tang, Y. Wang, X. Jiang, J. Wang, W. Bu, X. Zhang, Space-Selective Chemodynamic Therapy of CuFe_5_O_8_ Nanocubes for Implant-Related Infections, ACS Nano 14(10) (2020) 13391-13405.

[5] Z. Jin, Q. Jiang, B. Fang, B. Sun, The ArlR-MgrA regulatory cascade regulates PIA-dependent and protein-mediated biofilm formation in Rbf-dependent and Rbf-independent pathways, Int. J. Med. Microbiol. 309(2) (2019) 85-96.

[6] X. Qu, H. Yang, B. Jia, M. Wang, B. Yue, Y. Zheng, K. Dai, Zinc alloy-based bone internal fixation screw with antibacterial and anti-osteolytic properties, Bioact. Mater. 6(12) (2021) 4607-4624.

[7] X. Qu, H. Yang, B. Jia, Z. Yu, Y. Zheng, K. Dai, Biodegradable Zn-Cu alloys show antibacterial activity against MRSA bone infection by inhibiting pathogen adhesion and biofilm formation, Acta Biomater. 117 (2020) 400-417.

[8] B. Jia, H. Yang, Z. Zhang, X. Qu, X. Jia, Q. Wu, Y. Han, Y. Zheng, K. Dai, Biodegradable Zn-Sr alloy for bone regeneration in rat femoral condyle defect model: In vitro and in vivo studies, Bioact. Mater. 6(6) (2021) 1588-1604.

[9] X. Wei, W. Zhou, Z. Tang, H. Wu, Y. Liu, H. Dong, N. Wang, H. Huang, S. Bao, L. Shi, X. Li, Y. Zheng, Z. Guo, Magnesium surface-activated 3D printed porous PEEK scaffolds for in vivo osseointegration by promoting angiogenesis and osteogenesis, Bioact. Mater. 20 (2023) 16-28.

[10] F.A. Shah, M. Trobos, P. Thomsen, A. Palmquist, Commercially pure titanium (cp-Ti) versus titanium alloy (Ti6Al4V) materials as bone anchored implants - Is one truly better than the other?, Mater. Sci. Eng. C-Mater. Biol. Appl. 62 (2016) 960-966.

[11] D. Li, G. Fan, X. Huang, D. Juul Jensen, K. Miao, C. Xu, L. Geng, Y. Zhang, T. Yu, Enhanced strength in pure Ti via design of alternating coarse- and fine-grain layers, Acta Mater. 206 (2021) 1-13.

[12] K. Huang, F. Li, K. Yuan, Y. Yang, H. Chang, Y. Liang, X. Yan, J. Zhao, T. Tang, S. Yang, A MOF-armored zinc-peroxide nanotheranostic platform for eradicating drug resistant bacteria via image-guided and in situ activated photodynamic therapy, Appl. Mater. Today 28 (2022) 1-12.

[13] K.J. Qiu, Y. Liu, F.Y. Zhou, B.L. Wang, L. Li, Y.F. Zheng, Y.H. Liu, Microstructure, mechanical properties, castability and in vitro biocompatibility of Ti-Bi alloys developed for dental applications, Acta Biomater. 15 (2015) 254-265.

[14] I. Ghamarian, B. Hayes, P. Samimi, B.A. Welk, H.L. Fraser, P.C. Collins, Developing a phenomenological equation to predict yield strength from composition and microstructure in β processed Ti-6Al-4V, Mater. Sci. Eng. A-Struct. Mater. Prop. Microstruct. Process. 660 (2016) 172-180.

[15] Y. Sun, W. Zeng, Y. Han, Y. Zhao, G. Wang, M.S. Dargusch, P. Guo, Modeling the correlation between microstructure and the properties of the Ti–6Al–4V alloy based on an artificial neural network, Mater. Sci. Eng. A-Struct. Mater. Prop. Microstruct. Process. 528(29-30) (2011) 8757-8764.
